# Stability–Maneuverability Tradeoffs Provided Diverse Functional Opportunities to Shelled Cephalopods

**DOI:** 10.1093/iob/obac048

**Published:** 2022-11-09

**Authors:** David J Peterman, Kathleen A Ritterbush

**Affiliations:** Department of Geology and Geophysics, University of Utah, Salt Lake City, UT 84112, USA; Department of Geology and Geophysics, University of Utah, Salt Lake City, UT 84112, USA

## Abstract

Stability–maneuverability tradeoffs impose various constraints on aquatic locomotion. The fossil record houses a massive morphological dataset that documents how organisms have encountered these tradeoffs in an evolutionary framework. Externally shelled cephalopods (e.g., ammonoids and nautiloids) are excellent targets to study physical tradeoffs because they experimented with numerous conch morphologies during their long-lived evolutionary history (around 0.5 billion years). The tradeoff between hydrostatic stability and maneuverability was investigated with neutrally buoyant biomimetic models, engineered to have the same mass distributions computed for their once-living counterparts. Monitoring rocking behavior with 3D motion tracking reveals how stability influenced the life habits of these animals. Cephalopods with short body chambers and rapid whorl expansion (oxycones) more quickly attenuate rocking, while cephalopods with long body chambers (serpenticones and sphaerocones) had improved pitch maneuverability. Disparate conch morphologies presented broad functional opportunities to these animals, imposing several advantages and consequences across the morphospace. These animals navigated inescapable physical constraints enforced by conch geometry, illuminating key relationships between functional diversity and morphological disparity in aquatic ecosystems. Our modeling techniques correct for differences in material properties between physical models and those inferred for their living counterparts. This approach provides engineering solutions to the obstacles created by buoyancy, mass distributions, and moments of inertia, permitting more lifelike, free-swimming biomechanical models and aquatic robots.

## Introduction

Over 99% of all species to ever exist are now extinct (Novacek 2001). Consequently, numerous morphologies with unique biomechanical properties have also vanished. However, these disappearances do not always reflect suboptimal performance. These morphologies, familiar and otherworldly, are preserved in a vast library—the fossil record. Extinct organisms have much value in comparative biomechanics by supplying broader target morphologies and by adding an evolutionary (deep-time) context. Externally shelled cephalopods (ectocochleates) provide unique perspectives into aquatic locomotion. These animals evolved a buoyancy apparatus and jet propulsion to swim, becoming among the most complex and mobile group of mollusks. Today, few cephalopods manage buoyancy with an external, chambered conch. However, this architecture was common in the Paleozoic and Mesozoic. During this time, ectocochleate cephalopods (e.g., ammonoids, nautiloids, and others) experimented with immense variations in conch shape (Teichert et al. 1964; Saunders and Shapiro 1986; Saunders and Work 1996; Wright et al. 1996; Saunders et al. 2004; Korn and Klug 2012; Klug et al. 2016; Hoffmann, Slattery et al. 2021), preserving a highly disparate fossil record spanning around half a billion years (Kröger et al. 2011; Pohle et al. 2022). The morphological disparity of these extinct cephalopods likely reflects comparable differences in the functional constraints on their life habits. In many cases, the most fundamental swimming capabilities of these animals are poorly known, though decades of research have focused on addressing this issue (Trueman 1940; Denton 1974; Jacobs 1992; Jacobs and Chamberlain 1996; Westermann 1996, 1998; Klug and Korn 2004; Hoffmann et al. 2015; Naglik, Tajika et al. 2015). The current gap in knowledge is unfortunate because these animals were vital components of marine ecosystems for most of the Phanerozoic, and likely occupied diverse ecological niches across the globe (House 1981; Westermann 1998; Kruta et al. 2011; Korn et al. 2015; Tajika et al. 2018). Furthermore, a more detailed understanding of the selective pressures acting on these animals would provide key context to every mass extinction and numerous other environmental perturbations throughout the Phanerozoic. Recent advances in computer modeling and physical model construction allow the functional constraints of these animals to be investigated quantitatively, providing new insights into the life capabilities of these ecologically significant animals.

**Fig. 1:**
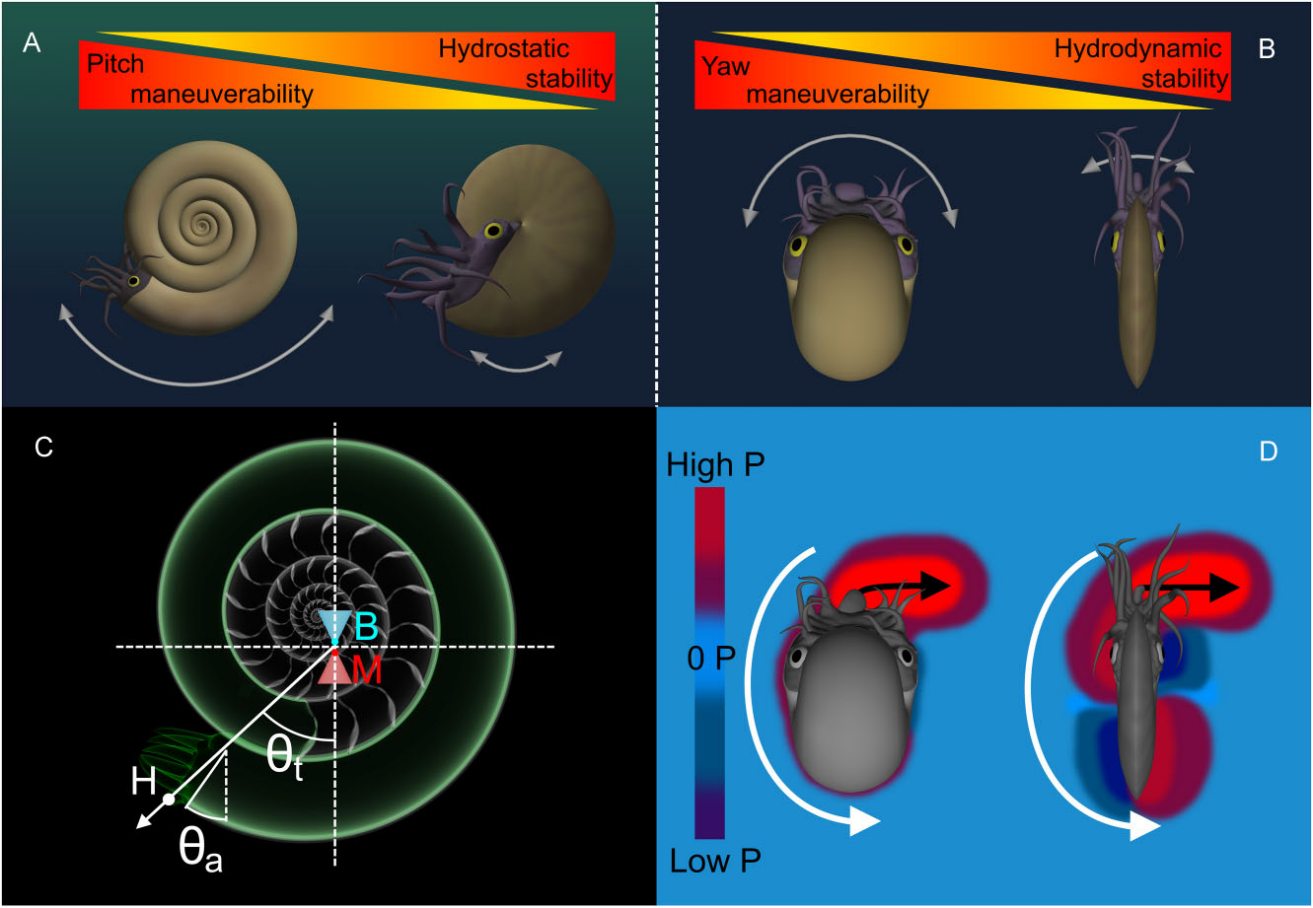
Hydrostatic and hydrodynamic properties of planispiral cephalopods. **A** Depiction of the tradeoff between hydrostatic stability and pitch maneuverability (lateral view). **B** Depiction of the tradeoff between hydrodynamic stability and yaw maneuverability (top view). Arrows denote the magnitude of rotation about the horizontal axis (**A**), and vertical axis (**B**). **C** Diagram of various hydrostatic properties (B = center of buoyancy, M = center of mass, H = source of jet thrust at the hyponome, θ_*t*_ = thrust angle, θ_a_ = apertural angle). Dashed lines represent the horizontal and vertical axis passing through the hydrostatic centers. **D** Theoretical diagram of pressures (P) experienced by an inflated and compressed conch shape during rotation (counterclockwise yaw rotation depicted). Black arrows denote jetting direction and white arrows indicate movement of the conch.

**Fig. 2:**
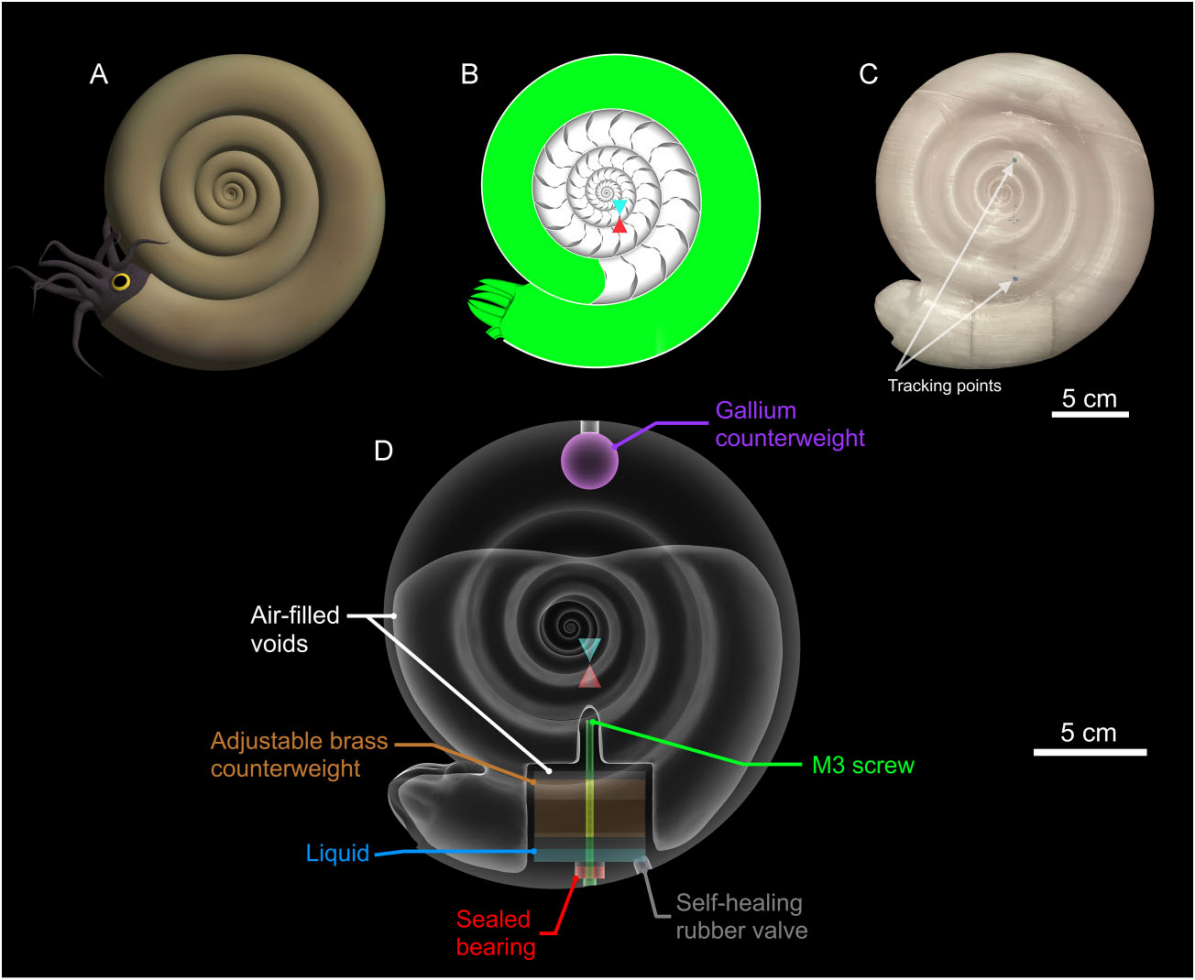
Construction of 3D-printed models. **A** Reconstruction of a serpenticone cephalopod (resembling the ammonoid, *Dactylioceras commune* with no ornament). **B** Virtual hydrostatic model of a serpenticone ammonoid, modified from Peterman and Ritterbush 2022). **C** Fully assembled, 3D-printed model with tracking points placed along the vertical axis passing through the hydrostatic centers (see Fig. 1). This model design allows rocking behavior to be recorded during 3D motion tracking. **D** Virtual model engineered with an adjustable counterweight system, allowing the total center of mass to be adjusted with sub-millimeter level accuracy. Each model component compensates for the differences in densities (color-coded) and internal shapes between the virtual hydrostatic model and the physical model. Additionally, the counterweight chamber allows for a range of motion—from hydrostatic inversion to the proper stability condition. The tips of the blue and red cones denote the centers of buoyancy and mass, respectively.

**Fig. 3:**
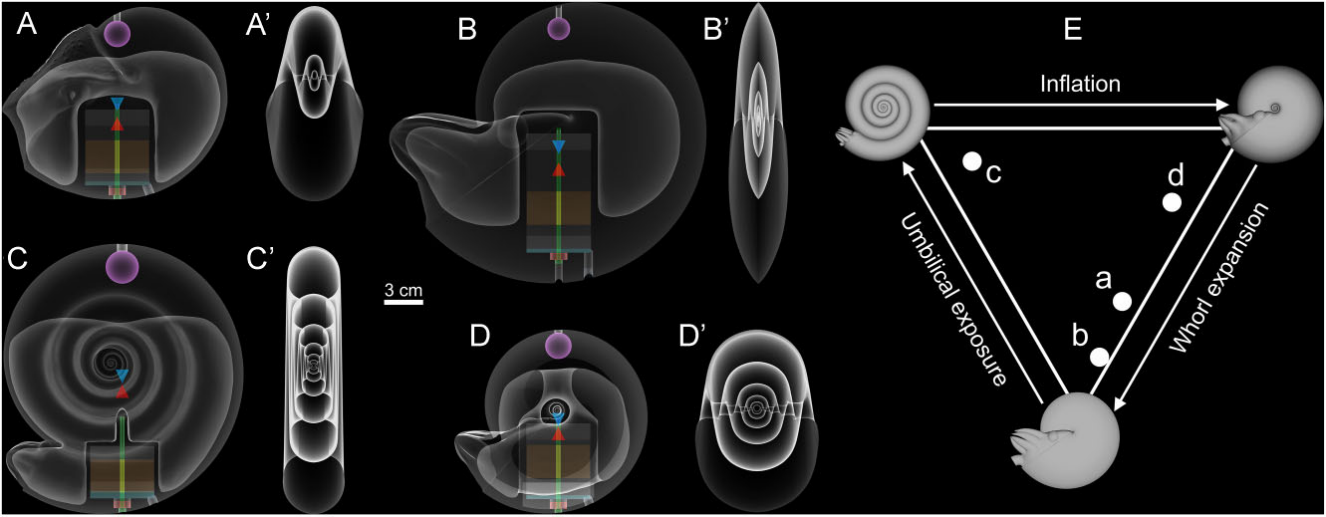
Schematic of physical models for each examined morphotype. **A** *Nautilus pompilius* (hydrostatic stability, *S_t_* = 0.043), **B** oxycone (*S_t_* = 0.068), **C** serpenticone (*S_t_* = 0.015), and **D** sphaerocone (*S_t_* = 0.006). Prime symbols (') denote the transverse, transparent view of external conchs. **E** Westermann Morphospace (Ritterbush and Bottjer 2012) showing the location of each examined conch shape. External shapes and hydrostatic stability indices are from Peterman and Ritterbush (2022). Colors correspond to the annotations on Fig. 1D. The tips of the blue and red cones denote the centers of buoyancy and mass, respectively.

Cephalopod conch shape (referring to the entire shell geometry) influences various syn vivo physical properties and functional constraints (Fig. 1). A conch's external shape constrains how the living animal interacts with surrounding water during locomotion (i.e., drag, lift, etc.; Trueman 1940; Denton 1974; Chamberlain 1976, 1981, 1993; Jacobs 1992; Jacobs et al. 1994; Jacobs and Chamberlain 1996; Naglik, Tajika, et al. 2015; Hebdon et al. 2020, 2021; Peterman, Hebdon et al. 2020; Peterman, Shell et al. 2020; Hebdon, Ritterbush et al. 2022), while internal morphology and coiling parameters influence hydrostatics (i.e., buoyancy, stability, directional efficiency of movement; Fig. 1C; Saunders and Shapiro 1986; Hoffmann et al. 2015; Peterman, Barton et al. 2019; Peterman, Mikami et al. 2020; Peterman, Yacobucci et al. 2020; Morón-Alfonso et al. 2021; Peterman et al. 2021; Peterman and Ritterbush 2022). Nautilids—the only living cephalopods with chambered, external conchs—are frequently used as models for the swimming capabilities and function of extinct taxa. However, the morphological and physiological disparity of extinct cephalopods are poorly represented by these extant animals, rendering them insufficient as analogues (Jacobs and Landman 1993; Klug and Lehmann 2015; Hoffmann, Slattery et al. 2021; Klug et al. 2021; Cherns et al. 2022). Alternatively, the morphological information preserved in the ectocochleate fossil record can be used to assess various biomechanical properties and functional constraints. To maintain a near neutrally buoyant condition, the proportions of void space in the chambered conch should manage organismal mass so that it is close to the mass of water displaced by the living animal. Conch coiling influences the distribution of organismal mass, largely due to the relative positions of the soft body occupying the body chamber and the air-filled chambers (Trueman 1940; Chamberlain 1981; Saunders and Shapiro 1986; Klug and Korn 2004). The static orientation these animals assumed during life can be determined when the total center of mass is vertically aligned under the center of buoyancy (Fig. 1C; Denton 1974; Saunders and Shapiro 1986; Klug and Korn 2004; Hoffmann et al. 2015; Naglik, Monnet et al. 2015). The living animals are more hydrostatically stable when these two centers are farther separated. Generally, planispiral conchs with larger body chamber proportions have lower hydrostatic stability (Raup 1967; Saunders and Shapiro 1986; Hoffmann et al. 2015; Lemanis et al. 2015; Peterman and Ritterbush 2022). Extant nautilids have short body chambers and are regarded as hydrostatically stable objects. These animals experience a strong restoring moment that realigns their hydrostatic centers after each jet pulse, which is demonstrated by their rocking behavior during locomotion. In the current study, we use biomimetic models (i.e., in terms of their mass distributions; Fig. 2) to investigate functional tradeoffs across the Westermann morphospace (Ritterbush and Bottjer 2012), an empirical morphospace where coiling parameters of planispiral cephalopods can be represented in a ternary diagram (Fig. 3E).

During the course of ectocochleate cephalopod evolution, we expect selection for different conch morphologies that function according to particular lifestyles. Traditionally, broad cephalopod morphogroups have been suggested to occupy particular life habits based on coiling parameters (Westermann 1996). While this scheme is speculative, it presents some testable hypotheses. According to Westermann (1996), oxycones (streamlined with high whorl expansion; Fig. 3B) were termed nektic while serpenticones (high umbilical exposure; Fig. 3C) and sphaerocones (high conch inflation; Fig. 3D) were termed planktic drifters and vertical migrants, respectively. Nektic life habits are supported for oxycones because they generally experience lower hydrodynamic drag (at higher Reynolds numbers) and have superior coasting efficiency (Jacobs 1992; Hebdon et al. 2021; Hebdon, Ritterbush et al. 2022; Peterman and Ritterbush 2022; Ritterbush and Hebdon 2022). However, a growing body of work is demonstrating that serpenticones and sphaerocones did not necessarily experience physical constraints that would have confined them to planktic life habits. Serpenticone conchs do not incur much more drag than oxycones of similar size (Hebdon, Ritterbush et al. 2022; Peterman and Ritterbush 2022). Sphaerocones have been determined to be more efficient moving at lower Reynolds numbers (Jacobs 1992; Jacobs et al. 1994; Hebdon et al. 2021; Hebdon, Ritterbush et al. 2022) and had superior mobility about the vertical axis (Peterman and Ritterbush 2022). Many shapes across the morphospace iteratively reappear (Bayer and McGhee 1984; Monnet et al. 2011; Monnet, Klug et al. 2015), suggesting positive adaptive value, even for morphotypes less hydrodynamically favorable than streamlined oxycones.

Physical tradeoffs between stability and maneuverability (Fig. 1A, B) considerably influence aquatic locomotion performances (Bayer 1982; Webb 1984, 2002; Fish 2002; Weihs 2002; Sefati et al. 2013; Fish and Holzman 2019; Peterman, Ciampaglio et al. 2019; Peterman and Ritterbush 2022). Different fish morphologies have been attributed to selection for various specializations (Webb 1984; e.g., acceleration, cruising, and maneuverability). Because fish can change shape and use various arrangements of fins during locomotion (Webb 1984, 2002; Weihs 1993; Lauder and Tangorra 2015) some fish can effectively eliminate certain tradeoffs (Sefati et al. 2013). Ectocochleates, however, are confined in rigid shells of a fixed shape, with one primary source of thrust (the hyponome; Fig. 1C). Therefore, the functional capabilities of these animals may have been more constrained by morphology compared to fishes. Furthermore, the detailed fossil record of ectocochleates and the distinct physical properties associated with conch shape suggest that these animals are excellent targets to study evolutionary biomechanics.

Among ectocochleates, compressed morphotypes (oxycones and serpenticones) had better coasting efficiency (hydrodynamic stability) while inflated morphotypes (sphaerocones) could more easily rotate about the vertical axis (yaw maneuverability; Fig. 1B, D; Bayer 1982; Peterman and Ritterbush 2022). In the current study, we investigate another physical tradeoff—between hydrostatic stability and maneuverability about the horizontal axis (pitch; Fig. 1A) for three near-endmembers of the Westermann morphospace and a model of an extant *Nautilus pompilius*. Rather than only depending upon external shape, hydrostatic stability always passively acts on these living animals, imparting some restoring moment that returns them to their proper static orientation (Peterman, Ciampaglio et al. 2019; Peterman, Shell et al. 2020). Higher stability can prevent unwanted rocking and allows a wider swath of jetting directions that result in movement. However, this condition may also prevent the living animals from modifying their own orientations. In the current study, we use 3D-printed models of disparate morphologies to impart the hydrostatic properties of their once-living counterparts (represented by theoretical morphologies to isolate variables). These models (Figs. 2 and 3; Supplementary Fig. S1) are engineered with adjustable counterweights to control the total mass distribution at the sub-millimeter scale. Low hydrostatic stability conditions have presented barriers to accurately reconstructing animals with biomimetic models. This new method allows hydrostatic experiments for morphotypes with low stability values, which represent the majority of the cephalopod morphospace (Trueman 1940; Chamberlain 1981; Saunders and Shapiro 1986; Peterman and Ritterbush 2022). Furthermore, this method also accounts for moments of inertia (rotational resistance), allowing the models to behave closer to the living animals in water. While hydrostatic stability has been quantified (or estimated) through various techniques (Trueman 1940; Chamberlain 1981; Saunders and Shapiro 1986; Jacobs and Chamberlain 1996; Okamoto 1996; Hoffmann et al. 2015; Tajika et al. 2015; Peterman, Barton et al. 2019; Peterman, Mikami et al. 2020; Morón-Alfonso et al. 2021), its relationship to locomotion and maneuverability is unclear. Using 3D motion tracking, we monitor hydrodynamic restoration for different morphologies in a chaotic, real-world setting. This approach was used to investigate fundamental constraints on swimming capabilities, including: (1) whether morphotypes with low hydrostatic stability values are sufficiently oriented in water, (2) the magnitude of hydrodynamic restoration for the full spectrum of stabilities experienced by planispiral cephalopods, and (3) the interaction of hydrostatics and hydrodynamics for disparate conch shapes. Ultimately, this approach is used to reevaluate cephalopod swimming capabilities and life habits across the planispiral morphospace by illuminating essential functional constraints.

## Methods

Physical models (Fig. 2C) were constructed with adjustable counterweights (Figs. 2D and 3; Supplementary Fig. S1) that allow their 3D mass distributions to be calibrated in water. These counterweights were used to impart the same hydrostatic stabilities inferred for their living counterparts. Additionally, the counterweight chamber simultaneously functions as a buoyancy adjustment device, allowing liquid to be injected through a self-healing rubber valve. These physical models were constructed in a virtual setting, then 3D-printed to investigate their kinematics with 3D motion tracking. Ultimately, these models serve as proxies for disparate cephalopod morphologies across the planispiral (Westermann) morphospace (Ritterbush and Bottjer 2012), permitting exploration of their relative physical constraints on syn vivo swimming capabilities.

### Virtual hydrostatic models

Virtual hydrostatic models, representing the living animals, were constructed in earlier studies. These models were created for disparate conch shapes across the planispiral morphospace (Ritterbush and Bottjer 2012), including three near-endmembers and an extant *Nautilus pompilius* (models from Peterman, Barton et al. 2019; Peterman and Ritterbush 2022). Three near-endmembers were chosen from ammonoid coiling parameters: oxycone (streamlined with high whorl expansion; e.g., *Sphenodiscus lobatus*; Fig. 3B), serpenticone (high umbilical exposure; e.g., *Dactylioceras commune*; Fig. 3C), and sphaerocone (high conch inflation; e.g., *Goniatites crenistra*; Fig. 3D). A CT-scanned *Nautilus pompilius* conch was morphed into each of these conch shapes to equalize the variables of septal shape, septal spacing, septal thickness, and shell thickness. Only body chamber lengths and conch coiling vary between these models, which most heavily influence hydrostatics (Saunders and Shapiro 1986; Hoffmann et al. 2015; Peterman and Ritterbush 2022). Since the behavior of extant nautilids are observable and well documented, a virtual model of *Nautilus pompilius* was also created in an earlier study (Peterman, Barton et al. 2019) to provide a point of comparison for the physical properties of extinct morphologies. Detailed methods of model construction and conch parameters are further described in earlier related studies (Peterman, Barton et al. 2019; Peterman and Ritterbush 2022).

### Physical model design

First, the external interfaces of each hydrostatic model (*Nautilus*, oxycone, serpenticone, and sphaerocone) were isolated and scaled to nearly identical volumes and masses (∼985 g; Supplementary Table S5). This value was arbitrarily chosen because it corresponds to a sphaerocone model of 15 cm conch diameter used in an earlier study (Peterman and Ritterbush 2022). Adjustable counterweights allow the total center of mass to be manipulated in water. By understanding the contribution of this component to the total mass distribution, the counterweight can be moved by some distance, which separates the total center of mass from the center of buoyancy, imparting the proper hydrostatic stability index. The counterweights in each model were designed to be capable of a range of motion, allowing hydrostatic inversion (up–side–down orientations) and having their proper stability imparted. This range of motion allowed a zero-stability condition to be calibrated in water (i.e., when the centers of buoyancy and mass coincide, and the model assumes no preferred orientation). Afterward, the counterweight must move the following distance (D) to impart the proper hydrostatic stability:

(1) \begin{eqnarray*}D\ = \ \frac{{{m}_{wd}}}{{{m}_{bc}}}{S}_t\sqrt[3]{{{V}_{wd}}}\end{eqnarray*}

Where *m_wd_* and *m_bc_* are the masses of the water displaced by the model and the adjustable brass counterweight, respectively. The mass of the water to be displaced by the physical model was computed from the volume of the external model using MeshLab (Cignoni et al. 2008). The product of the hydrostatic stability index (*S_t_*) and the cube root of the volume of water displaced (*V_wd_*) is equal to the distance between the centers of buoyancy and mass. Stability indices for each investigated morphotype were computed in Peterman and Ritterbush (2022), and are listed in Supplementary Table S1.

A chamber in the model was constructed that accommodates this computed range of motion (D) for the adjustable counterweight, with some leeway in either vertical direction. Virtual models were created for each component of the adjustable counterweight mechanism with the 3D modeling program, Blender (Blender Online Community 2017), including: a brass hex rod counterweight tapped with 0.5 mm pitch threads, an M3 screw, a self-healing rubber valve for buoyancy adjustment, and a sealed bearing that allows the screw to turn while keeping the adjustment chamber watertight (Fig. 2D; Supplementary Fig. S1). The centers of these models were aligned with the vertical axis passing through the center of buoyancy (B). This center is equal to the center of volume of water displaced, and was computed from the external model in MeshLab (Cignoni et al. 2008). The stability adjustment mechanism was placed underneath the model (low *z* direction) to allow access to the adjustment screw. To offset the location of this mechanism, a stationary counterweight (made of Gallium) was positioned at the opposite end of the model (high *z* direction; Fig. 2D). The initial models were created by placing the virtual model of the adjustable brass counterweight in its upper location (ideally producing zero stability).

Afterward, the required volume of the 3D-printed thermoplastic (PETG—polyethylene terephthalate glycol) was computed from the mass required to produce a neutrally buoyant condition (accounting for ∼2–5 g of liquid to be inserted into the internal chamber to fine-tune buoyancy; Fig. 2D). This PETG mass was computed by subtracting all other model components (brass counterweight, gallium counterweight, M3 screw, sealed bearing, self-healing rubber, and chamber liquid; Supplementary Table S5) from the mass of the water displaced. The center of mass of the PETG virtual model was iteratively adjusted, while holding volume constant, to correct for the influence of all other model components on the total center of mass. The target PETG center of mass was computed with the following equation:

(2) \begin{eqnarray*}{D}_{{\rm{PETG}}} = \frac{{M\mathop \sum \nolimits_{i = 1}^n {m}_i - \ \mathop \sum \nolimits_{i = 1}^n ({D}_i{m}_i)}}{{\left( {{m}_{{\rm{PETG}}}} \right)}}\ \end{eqnarray*}

Where *D*_PETG_ is the location of the PETG 3D-printed model's center of mass, measured from an arbitrary datum in each principal direction. *M* is the total center of mass in a particular principal direction (originally modeled to equal the center of buoyancy at the 3D origin), *m_i_* is the mass of each model component, *D_i_* is the local center of mass of each model component in a particular principal direction, and *m*_PETG_ is the mass of the PETG required for a neutrally buoyant condition. See Supplementary Tables S5 and S6 for a list of model components and measurements.

The hydrostatic stability index for the virtual model was computed with the following equation:

(3) \begin{eqnarray*}{\rm{\ }}{S}_t = \ \frac{{\sqrt {{{( {{B}_x - {M}_x})}}^2 + {{( {{B}_y - {M}_y})}}^2\ + \ {{( {{B}_z - {M}_z})}}^2} }}{{\sqrt[3]{{{V}_{wd}}}}}\end{eqnarray*}

Where the subscripts correspond to the *x*, *y*, and *z* components of the centers of buoyancy (*B*) and mass (*M*), and *V_wd_* is the volume of water displaced. Note that the numerator is equal to the 3D distance between the centers of buoyancy and mass. The total center of mass (*M*) was computed from the local centers of mass for each model component:

(4) \begin{eqnarray*}M\ = \frac{{\sum \left( {L*{m}_o} \right)}}{{\sum {m}_o}}\ \end{eqnarray*}

Where *M* is the total center of mass in a principal direction, *L* is the center of mass of a single object measured with respect to an arbitrary datum in each principal direction, and *m*_o_ is the mass of each object with unique density. This equation was used in the *x*, *y*, and *z* directions to compute the 3D coordinate position of the center of mass. The local centers of mass for each material of unique density (PETG, brass counterweights, gallium counterweights, screws, bearings, self-healing rubber, and chamber liquid; Fig. 2D) were computed in MeshLab (Cignoni et al. 2008). The center of buoyancy (*B*) is equal to the center of volume of water displaced by the model. This volume was constructed by isolating the external interface of the model and computing its center in MeshLab (Cignoni et al. 2008).

The final models (Fig. 3) have variable 3D distributions of each component (Supplementary Table S6), but are capable of fine-tuning buoyancy and hydrostatic stability in a physical setting. These models and example footage are stored in an online repository (Dataset S1; DOI: 10.5281/zenodo.6316035).

### Physical model construction and assembly

The adjustable counterweights were machined from a 1″ wide (∼25.4 mm) brass hex rod, cut into pieces around 48 mm long, and tapped to produce 0.5 mm pitch threads. Each counterweight had slightly different total lengths, which were measured with digital calipers and accounted for in each virtual model. Each physical model was 3D-printed with an Ultimaker S5 3D printer in natural (clear) PETG filament with solid (100%) infill (Supplementary Fig. S1). These models were printed in three parts, sliced with flat surfaces to improve adhesion to the 3D printer buildplate. Part 1 consisted of the adjustable counterweight chamber and a separate air-filled, watertight void (Fig. 2) that was enclosed during printing. Part 2 was printed solid, except for a void with the same volume as the gallium counterweight. This metal was used for the secondary counterweight because it melts at around 30°C, allowing the required amount to be injected into the model with a syringe. Part 3 served as a cover for the adjustable counterweight chamber (Supplementary Fig. S1). This part includes a hole for the M3 screw, and a space to insert the sealed bearing. The bearing was glued in place with low viscosity cyanoacrylate to create a watertight seal. The M3 screw head was larger than the bearing preventing it from pulling through. A small 3D-printed nut was screwed on to the threaded side until it plugged the central hole in the bearing, twisting with pliers to ensure a tight fit. Afterward, the threaded brass counterweight was set onto the screw with minor amounts of white lithium grease to reduce friction. Each of the three parts were fit together and chemically welded with 100% dichloromethane to produce a watertight seal. Finally, the self-healing rubber valve was fit into the allotted space and glued with high viscosity cyanoacrylate. Slight differences in PETG mass (i.e., density differences due to gcode printing paths and/or different batches of filament) were corrected by adjusting the final mass of gallium. Model buoyancies were calibrated in 32.2°C water (same as the experimental setting) so that they were positively buoyant until placing a 3 g washer on top caused them to sink (i.e., between 0 and 3 g of internal liquid required for neutral buoyancy). Tracking points (Fig. 2C) were placed on each model in dark, contrasting colors to monitor hydrodynamic restoration and stability with 3D motion tracking.

### Rocking experiments

Rocking behavior (hydrodynamic restoration and stability) was investigated for the physical models in a 5-foot-deep section of a pool at the University of Utah's Crimson Lagoon. First, buoyancy was calibrated by injecting small amounts of liquid (∼3 g) through a self-healing rubber valve. Perfect neutral buoyancy can only be approached, so models experienced some vertical movement over long timespans (∼15–30 s). Next, counterweights were adjusted so that the model assumed no static orientation (indicating that the centers of buoyancy and mass coincided). Afterward, the counterweight was lowered by the proper amount for each model (Supplementary Table S1) by computing the number of screw turns to move a specified distance:

(5) \begin{eqnarray*}R\ = \ \frac{{\left( {\frac{{{m}_{wd}}}{{{m}_{bc}}}{S}_t\sqrt[3]{{{V}_{wd}}}} \right)}}{{{P}_t}}\end{eqnarray*}

Where *R* is the number of revolutions the screw must take (i.e., screw turns) to impart the proper hydrostatic stability. Note that the product of the hydrostatic stability index (*S_t_*) and cube root of the volume of water displaced (*V_wd_*) is equal to the distance between the centers of buoyancy and mass. The threaded brass counterweight in the model must move a greater distance, according to the ratio of the mass of water displaced (*m_wd_*) and the mass of the brass counterweight (m_*bc*_). This aspect of the model design allows very small distances between the hydrostatic centers to be imparted since the range of movement is magnified according to this ratio. Each M3 screw passing through the brass counterweight had a thread pitch (*P_t_*) of 0.5 mm/revolution, which moved the counterweight by this specified distance for each screw turn.

**Fig. 4:**
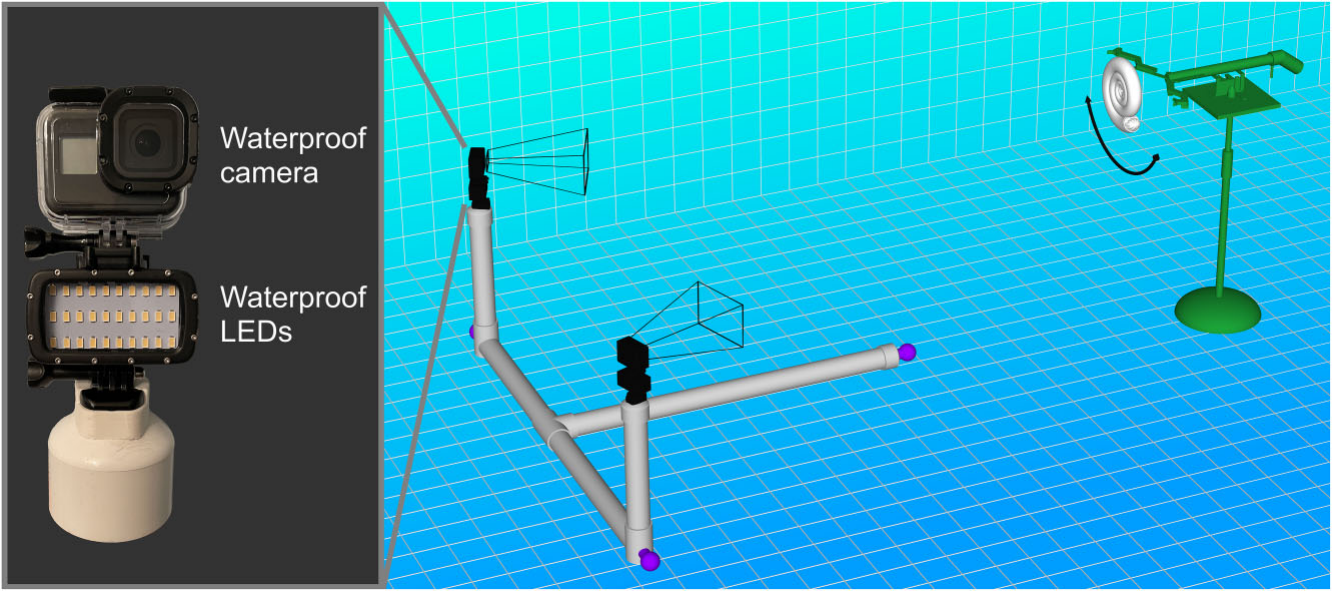
Schematic of the 3D motion tracking setup. Two waterproof cameras and LEDs are attached to a PVC frame (gray), with three counterweights on each end (purple). Each model was released with a submersible release mechanism (green) to monitor hydrodynamic restoration with 3D motion tracking.

A spring-loaded release mechanism was constructed (Fig. 4) that was capable of grabbing each model with extendable claws. The upper portion sat on a hinge, allowing each model to be consistently rotated. A 3D-printed wedge only allowed ∼55° of rotation from the static orientation assumed by each model. After releasing the trigger, both claws retracted backward and rotated away from the model (preventing the models from bumping into the device, and minimizing wake with low-profile shapes). A heavy, cast iron stand prevented unwanted movement in the submerged release mechanism. A total of 15 trials were performed for each of the four models in both release directions (starting with either aperture backward or aperture forward release). The counterweights were reset after every five trials to examine whether or not error was introduced when determining the zero-stability condition.

### 3D motion tracking

Rocking behavior was monitored with two waterproof cameras on a submersible camera rig (Fig. 4). Footage was recorded with two GoPro Hero 8 Black cameras at 4k resolution, 24 (23.975) frames/s, and linear fields of view. Motion tracking was performed with DLTdv8 (Hedrick 2008) to automatically identify the pixel coordinates of the upper and lower tracking points in each frame. Wand calibration was performed with easyWand5 (Theriault et al. 2014) to transform these pixel locations into 3D coordinates using the known distance between the two tracking points (Supplementary Table S8). Tracking points were monitored until they were obscured from view (yaw rotation), or until they approached boundary features (pool bottom or water surface).

After releasing each model, their rocking behaviors were reminiscent of the same motion a pendulum experiences. However, because these models were free to rotate in a 3D setting, they behaved more like spherical pendulums, experiencing nonplanar oscillation due to sensitive initial conditions and random currents in the pool. These out of plane rotations (yaw and roll) during rocking were corrected with a rotation matrix to confine all 3D points to a 2D plane. Otherwise, only the apparent angle would be recorded, which is lower than the true relative angle between each tracking point (θ_corr_):

(6) \begin{eqnarray*}{\theta }_{{\rm{corr}}} = \ \frac{{\left| {{x}_u - {x}_l} \right|{{\cot }}^{ - 1}\left( {\frac{{{z}_u - {z}_l}}{{\sqrt {{{\left( {{y}_u - {y}_l} \right)}}^2 + {{\left( {{x}_u - {x}_l} \right)}}^2} }}} \right)}}{{\left( {{x}_u - {x}_l} \right)}}*\frac{{180}}{\pi }\end{eqnarray*}

Where *x*, *y*, and *z* refer to each 3D component of the upper (*u*) and lower (*l*) tracking points. This equation transforms the *x*, *y*, and *z* points into angles displaced from the static equilibrium orientation of 0 degrees. Afterward, positive numbers were assigned apertures tilted upward from their equilibrium orientation, while negative numbers were assigned to apertures tilted downward from their equilibrium orientation. Rocking behavior in this setting was best captured with a harmonic oscillation function. The angle displaced from the static orientation through time (θ*_d_*) was fit with the following equation using the curve fitting toolbox in MATLAB:

(7) \begin{eqnarray*}{\theta }_d\left( t \right)\ = \ {\theta }_0{e}^{ - \gamma t}\cos \left( {\omega t} \right)\end{eqnarray*}

Where θ_0_ is starting angle rotated from the static orientation. The target starting angle in the current experiments was 55° for all models, with some deviation due to human error. The damping coefficient (γ) governs how quickly the amplitude of oscillations decay (similar to an exponential decay coefficient). Angular frequency (ω) controls the frequency of the fit data, with larger values causing more frequent oscillations. Additionally, due to underdamped harmonic oscillation, the wavelengths of each cycle increase through time.

## Results

**Fig. 5:**
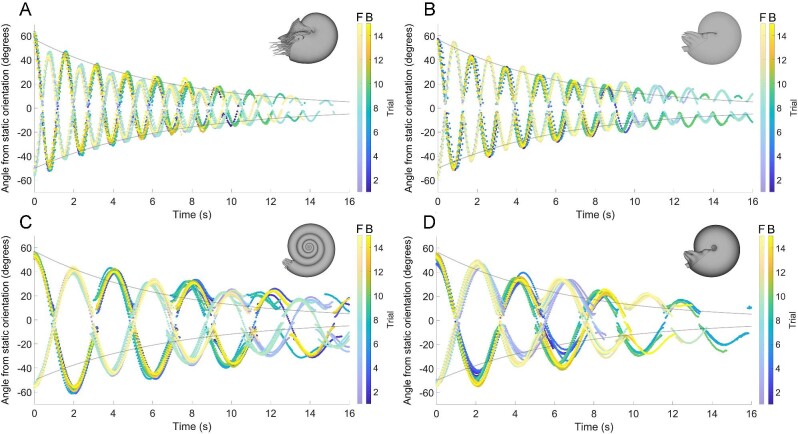
Rocking behavior computed from 3D motion tracking. Each model experiences underdamped harmonic oscillation, where positive angles correspond to apertures tilted upward from their static orientations and negative angles denote downwardly tilted apertures. **A** *Nautilus pompilius*, **B** oxycone, **C** serpenticone, and **D** sphaerocone. Points are color-coded by trial (F = aperture forward initial release, and B = aperture backward initial release). The gray lines on each panel denote the damping behavior of the *Nautilus pompilius* model (i.e., only damping coefficients considered, while setting angular frequency to zero).

The 3D-printed models are composed of materials that differ in densities, volumes, and mass distributions compared to the living animals. However, the models correct for these differences with a series of counterweights (Fig. 2), producing a condition close to neutral buoyancy while simultaneously imparting the same total mass distribution inferred for the living animals (representing theoretical morphologies; Supplementary Table S1; see Methods). Creating each model with nearly identical volumes (∼985 cm^3^) allows comparisons between rotational kinematics because the driving forces (gravity acting on model mass, and buoyancy) are nearly identical. Each of the investigated morphologies (*Nautilus pompilius*, oxycone, serpenticone, and sphaerocone; Fig. 3) restore themselves in water according to their differing hydrostatic stability indices (Supplementary Table S1) and external shapes (hydrodynamic drag). Additionally, moments of inertia (rotational resistance) differ between each morphology because this property depends on how mass is distributed relative to some rotational axis. Disparities between the moments of inertia for the 3D-printed models and virtual models representing the living animals are low (between ∼−3% and ∼11%; Supplementary Table S2; Supplementary Fig. S2). Furthermore, the differences in rotational kinematics were investigated by computing oscillation periods from a theoretical scenario where hydrodynamic drag is ignored (i.e., pendulous rocking in a vacuum). Under this scenario, differences in oscillation period attributed only to moments of inertia and hydrostatic stability range from −1.35% to 5.7% between the 3D-printed models and their cephalopod counterparts (Supplementary Table S2; Supplementary Fig. S2). After rotating each model ∼55° in each direction, they underwent pendulum-like rocking motions. Specifically, these models all experienced underdamped harmonic motion (i.e., oscillation with attenuation in amplitude and frequency). Damping coefficients (γ) and angular frequency (ω) were used to assess rotational kinematics (Equation 7). High damping coefficients correspond to amplitudes that decay more quickly, while high angular frequencies denote quicker cycles of oscillation. Both of these values are directly proportional to the magnitude of the restoring moment and hydrostatic stability. Additionally, these models experienced other forms of movement superimposed with their rocking behavior: yaw, roll, lateral translation (due to ambient water currents), and vertical translation (due to slight positive or negative buoyancies in the model; Supplementary Figs. S3 and S4). Despite these somewhat chaotic conditions, the rotational kinematics computed from 3D motion tracking (see Methods) are strikingly regular (Fig. 5). Model buoyancies were readjusted after every five trials, along with counterweight positions. The correct hydrostatic stability was imparted in each model by making them have zero stability (i.e., coincidence of the centers of buoyancy and mass, when no preferred orientation is assumed), then computing the screw turns to lower the threaded counterweight inside each model (Supplementary Table S1; Supplementary Fig. S1). The models could be inverted easily within 0.25 screw turns, suggesting that the total center of mass could reliably be imparted within ∼30 microns (see Equation 5). Recalibrations of the counterweights (Fig. 2; Supplementary Fig. S1) were intended to assess human inconsistencies in identifying the zero-stability condition. Despite some variation in variables fit to Equation 7 (Supplementary Fig. S5; Supplementary Table S3), the rotational kinematics of each investigated morphology are distinct from one another. Differences in kinematics within each model and between counterweight resets are likely due to subtle differences in identifying the zero-stability condition (when the model assumed no preferred orientation) before lowering the counterweight with the proper amount of screw turns (Equation 5). Additionally, deviation in the starting angle (θ_0_) from the target value of 55° caused small phase-shifts in oscillations (Supplementary Table S3), and differing trial durations influenced the fit parameters. However, when considering all 15 trials, the variables of Equation 7 (damping coefficient γ and angular frequency ω) can be statistically distinguished between each investigated morphology based on non-overlapping 95% confidence intervals (Supplementary Table S4). For each morphotype, differences in damping coefficients and angular frequencies between forward and backward release directions are minor, indicated by mostly overlapping 95% confidence intervals. Therefore, hydrostatic stability, gross hydrodynamic drag, and moments of inertia are most influential on rotational kinematics.

### Hydrodynamic restoration and hydrostatic stability

**Fig. 6:**
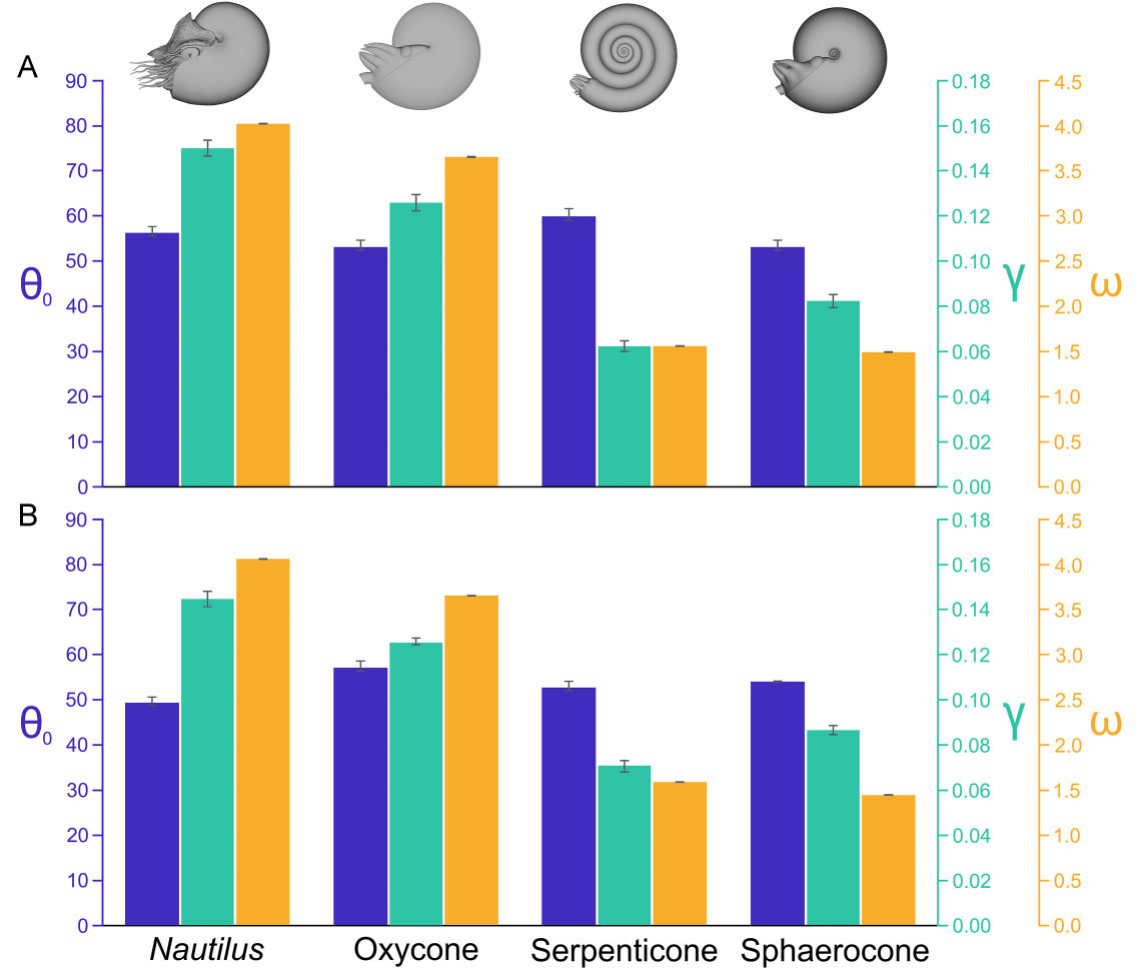
Harmonic oscillation variables fit to the kinematics of each model. **A** Aperture backward initial release, **B** aperture forward initial release. Variables of Equation 7 were fit in MATLAB (θ_0_ = starting angle in degrees, purple; γ = damping coefficient in s^−1^, teal; and ω = angular frequency in rad/s, yellow). Higher damping coefficients return the models back to their static orientations more quickly, while high angular frequencies yield more oscillations per unit time. Error bars denote 95% confidence intervals. Bar colors correspond to the colors of the axes.

Each of the examined morphologies can be classified into two groups: (1) hydrostatically stable (*Nautilus pompilius* and oxycone), and (2) hydrostatically unstable (serpenticone and sphaerocone). Hydrostatic stability indices (Supplementary Table S1) generally govern oscillation behavior in these groups. Stable morphotypes (Fig. 5A, B) experience quicker oscillations (higher angular frequencies) and quicker hydrodynamic restoration (higher damping coefficients). Unstable morphotypes (Fig. 5C, D) experience about half the damping and angular frequency compared to the stable morphotypes (Fig. 6).

**Fig. 7:**
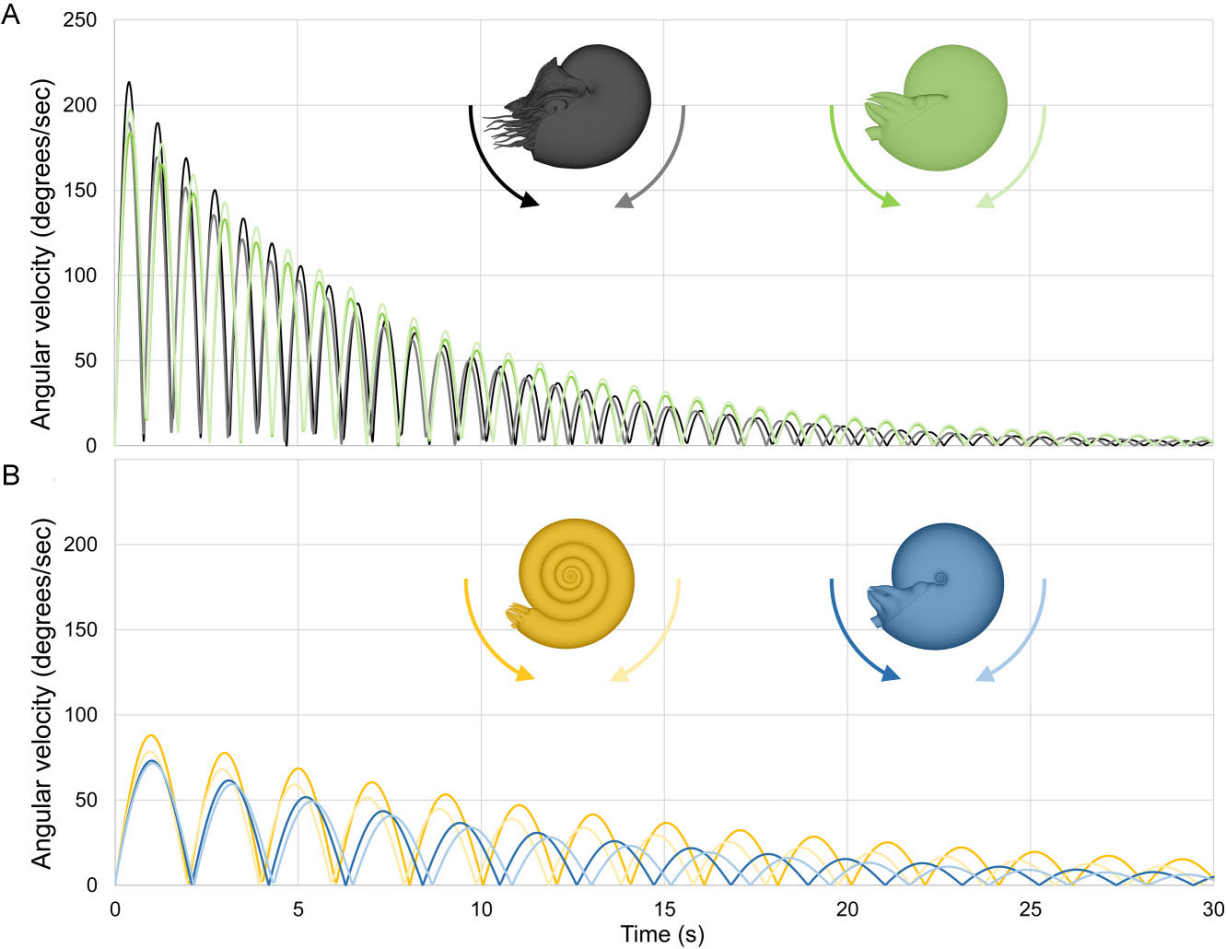
Angular velocities during hydrodynamic restoration. Values were computed from taking the time derivative of data fit to Equation 7. **A** Hydrostatically stable morphotypes (*Nautilus pompilius* and oxycone), **B** hydrostatically unstable morphotypes (serpenticone and sphaerocone). Colors indicate the model while arrows and their colors denote the initial release direction.

Higher stability attenuates rocking behavior and returns the cephalopod models back to their static orientations more quickly, and therefore, influences rotational speed (Fig. 7). That is, in addition to reaching lower rocking thresholds more slowly (Supplementary Fig. S6; Supplementary Table S7), the less stable morphotypes experience considerably lower angular velocities during restoration (Fig. 7).

While damping and angular frequency are generally proportionate to hydrostatic stability (i.e., separation between the hydrostatic centers), shape seems to obscure this relationship. Counterintuitively, the oxycone model which is around 1.6 times more hydrostatically stable (Supplementary Table S1) than *Nautilus* experiences lower damping coefficients and angular frequencies (Fig. 5). This is likely due to the sharp keel and streamlined shape encountering lower hydrodynamic drag (at the examined scale and velocities). By holding volume constant between each model, the moment of inertia is higher for more compressed shapes because more mass is distributed farther from the horizontal rotational axis. While the oxycone has a higher moment of inertia, it also has a longer lever arm (distance between hydrostatic centers; Supplementary Table S1). These differences compensate for each other, yielding similar oscillation periods when ignoring drag (i.e., in a theoretical vacuum where buoyancy is still somehow represented; Supplementary Fig. S2). The similarity in computed periods between the oxycone and *Nautilus* model suggests that hydrodynamic drag is slightly obscuring this relationship (a complex, transient property for rotational movement). The differences in soft body shape between the two models may also contribute to differences in drag (note that the *Nautilus* model has its tentacles smoothed away, but still has its characteristic hood; Fig. 3A). The sphaerocone also follows a trend not entirely related to hydrostatic stability, experiencing more rapid damping than the serpenticone (Figs. 5 and 6), despite having 40% of its hydrostatic stability index (Supplementary Table S1). The angular frequency of the sphaerocone, however, is slightly lower than the serpenticone (Fig. 6; Supplementary Table S4), which causes more gentle oscillations at longer time steps (especially for the projected data, beyond ∼12 s; Fig. 7). The sphaerocone has a lower moment of inertia and lever arm (distance between hydrostatic centers) compared to the serpenticone. These compensating differences also yield similar computed oscillation periods between these less stable models (in the same theoretical vacuum where buoyancy still exists; Supplementary Fig. S2). Both of these comparisons suggest that hydrodynamic drag attenuates rocking for more laterally inflated morphologies, although it plays a lesser role compared to the range of hydrostatic stabilities displayed by planispirals.

### Test cases to explore biomechanical tradeoffs

**Fig. 8:**
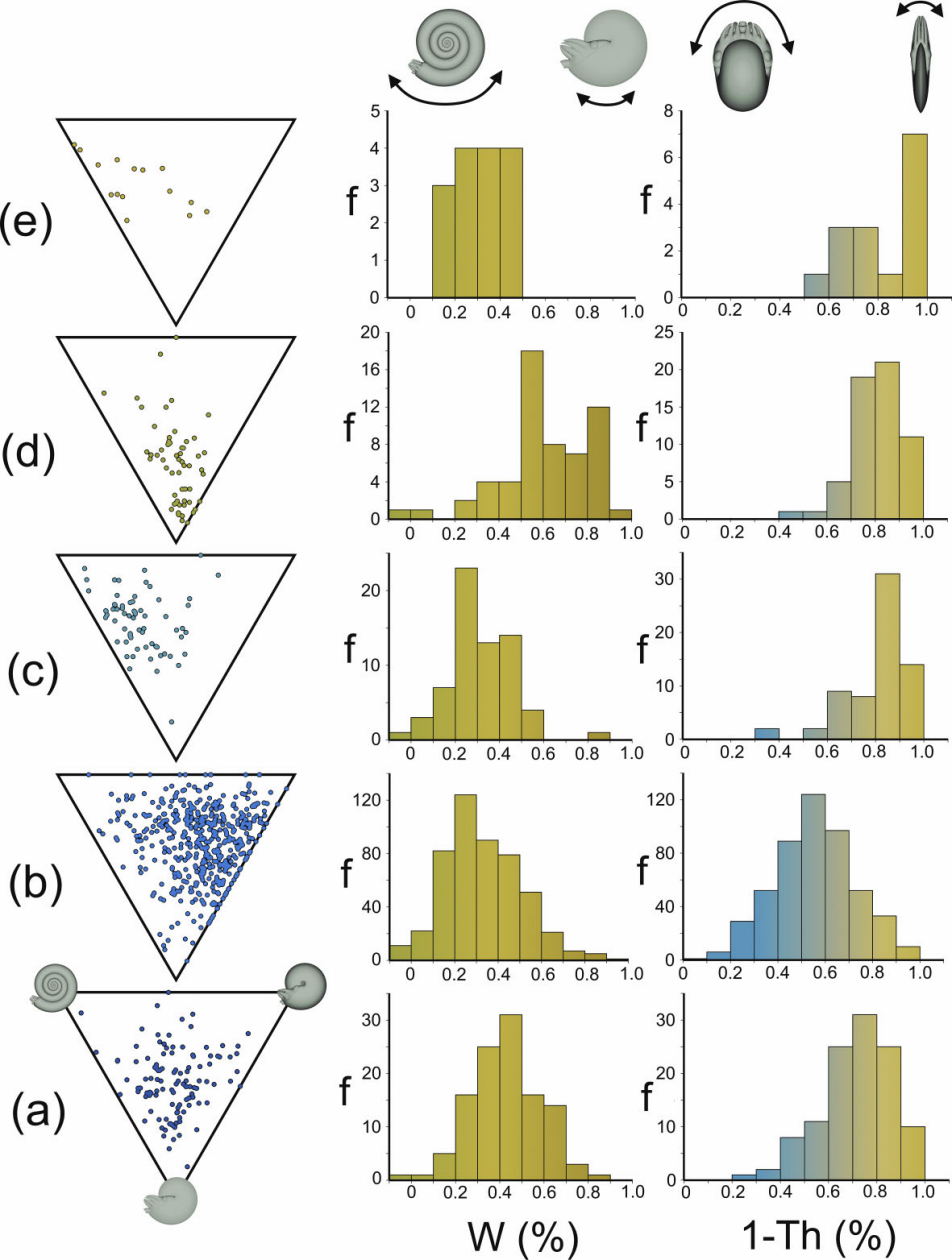
Coiling parameters used as a proxy for stability–maneuverability tradeoffs among Paleozoic ammonoid orders. **A** Agoniatitida, **B** Goniatitida, **C** Clymeniida, **D** Prolecanitida, **E** Ceratitida. Westermann morphospace plots, and histograms showing frequencies (f) of two conch parameters. Whorl expansion (W) is used as a proxy for hydrostatic stability (high values correspond to stable ammonoids while low values correspond to ammonoids with higher pitch maneuverability). The compression ratio (1-Th) is used as a proxy for hydrodynamic stability (high values corresponding to ammonoids with high coasting efficiency and course stabilization and low values corresponding to ammonoids with better yaw maneuverability; based on experiments with biomimetic cephalopod robots, Peterman and Ritterbush 2022). Original dataset recorded by Whalen et al. (2020).

**Fig. 9:**
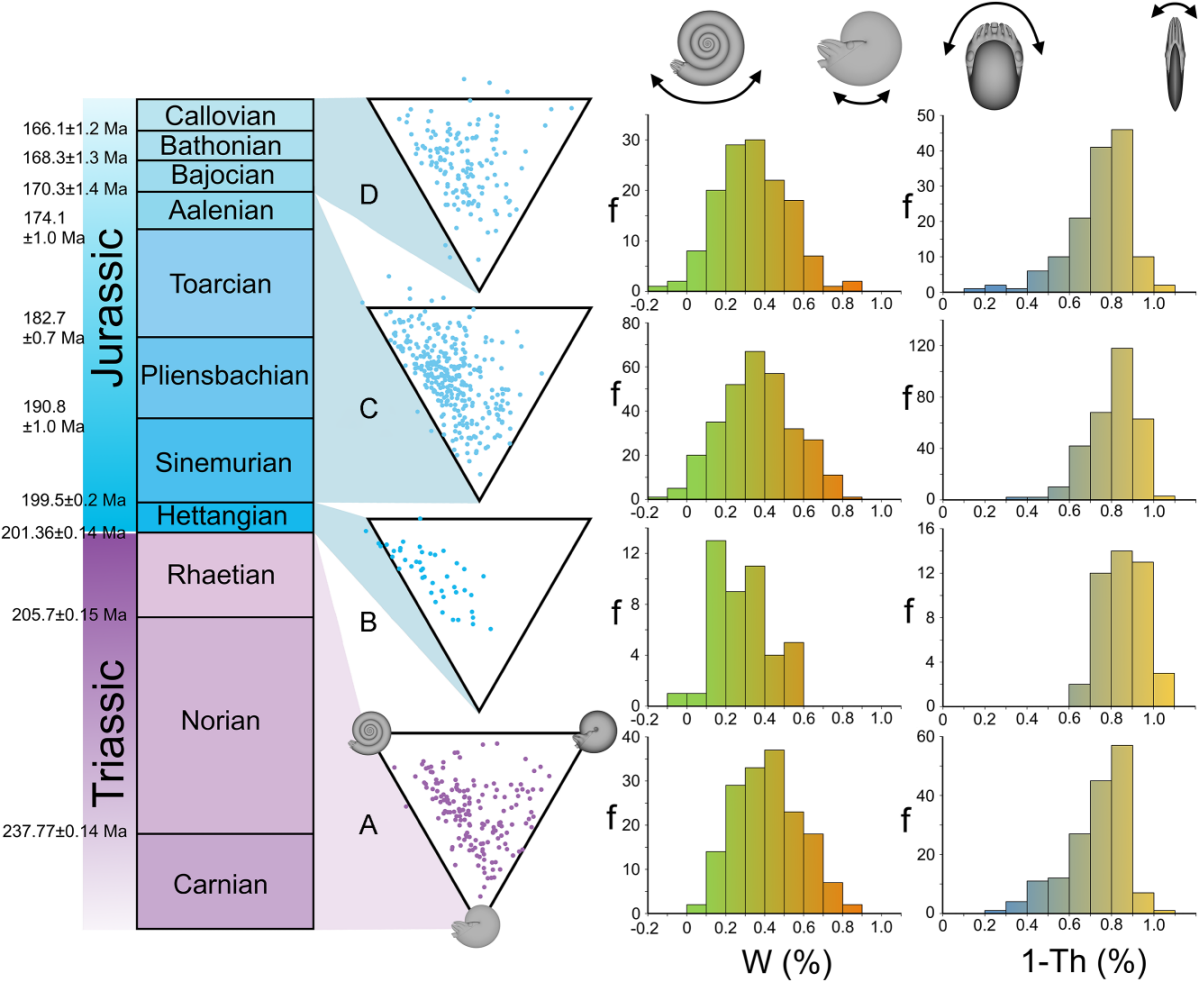
Coiling parameters used as a proxy for stability–maneuverability tradeoffs among Late Triassic to Middle Jurassic ammonoids. **A** Carnian-Rhaetian “pre-extinction” ammonoid faunas. **B** Hettangian ammonoid fauna representing the “aftermath” of the Triassic–Jurassic extinction. **C** Sinemurian–Aalenian “post-extinction” ammonoid faunas. **D** Bajocian–Callovian ammonoid faunas after recovering from the mass extinction event. Original conch measurements and designations are from Smith et al. (2014). These data were replotted in Westermann morphospace, and with histograms showing frequencies (f) of two conch parameters. Whorl expansion (W) is used as a proxy for hydrostatic stability (high values correspond to stable ammonoids while low values correspond to ammonoids with higher pitch maneuverability). The compression ratio (1-Th) is used as a proxy for hydrodynamic stability (high values corresponding to ammonoids with high coasting efficiency and course stabilization and low values corresponding to ammonoids with better yaw maneuverability; based on experiments with biomimetic cephalopod robots, Peterman and Ritterbush 2022).

The current experiments, and previous experiments with biomimetic robots (Peterman and Ritterbush 2022), demonstrate that conch morphology is closely tied to function, involving several tradeoffs between stability and maneuverability. We propose a scheme where conch coiling parameters are used as proxies for stability–maneuverability tradeoffs, falling on two continuums: whorl expansion ratio (W) is used as a proxy for hydrostatic stability (where lower values represent higher pitch maneuverability), and compression ratio (1-Th; inverse of thickness ratio) is used as a proxy for hydrodynamic stability (where lower values represent higher yaw maneuverability). We apply these proxies to two test cases: (1) comparing conch morphology between five ammonoid orders in the Paleozoic (Fig. 8; dataset from Whalen et al. 2020) and (2) comparing ammonoid conch morphology across the Late Triassic to Middle Jurassic (Fig. 9; dataset from Smith et al. 2014).

The frequency distributions of these two properties do not closely follow a normal distribution. Therefore, a Dunn's test was used to investigate whether or not distributions were statistically different for each combination of Paleozoic ammonoid orders (Supplementary Tables S9 and S10), and a Wilcoxon rank sum test was used to compare differences across Late Triassic to Mid Jurassic age bins (Supplementary Table S11). Nearly all conch coiling parameters between each Paleozoic ammonoid order can be statistically distinguished from each other at the α = 0.05 level. The Clymeniida and Ceratitida are most similar, and could not be distinguished in any coiling parameter (Supplementary Table S10). All other combinations could be distinguished by at least two coiling parameters (i.e., whorl expansion, conch compression, or umbilical exposure; Supplementary Table S10). Changes in coiling parameters between each stage of the Late Triassic to Middle Jurassic are more subtle. Compression ratio (1-Th) and umbilical exposure (U) significantly change across the Triassic–Jurassic boundary at the stage level of this dataset (examining the Rhaetian and Hettangian). When comparing the entire Triassic and Jurassic portions of the dataset, each coiling parameter changes, with significance at the α = 0.05 level (Supplementary Table S11).

## Discussion

### Hydrostatic stability vs. pitch maneuverability

The vast majority of planispiral cephalopods are much less hydrostatically stable than extant *Nautilus* (Trueman 1940; Raup 1967; Peterman and Ritterbush 2022). Virtual, hydrostatic models representing near-endmembers of the Westermann morphospace (Ritterbush and Bottjer 2012; oxycone, serpenticone, and sphaerocone) and a model of extant *Nautilus* demonstrate that these morphologies have considerable differences in hydrostatic stability, spanning over an order of magnitude (Supplementary Table S1). Orthocone (straight-shelled) cephalopods probably represent the endmember for high stability among ectocochleates, with stability values another order of magnitude higher than *Nautilus* (Peterman, Barton et al. 2019; Peterman, Ciampaglio et al. 2019; *S_t_* ∼0.5, specifically for those lacking internal counterweights like cameral deposits). Under this condition, orthocones quickly reach a static vertical orientation without rocking (overdamped harmonic motion). In contrast, the full range of stability values among planispirals produce underdamped harmonic oscillation (pendulum-like rocking). The mass distributions produced by each disparate conch geometry directly controls hydrodynamic restoration (i.e., when the centers of buoyancy and mass are displaced from vertical alignment). Analyses of rotational kinematics with 3D-printed hydrostatic models highlight a tradeoff between hydrostatic stability and pitch maneuverability. These experiments demonstrate that conch morphology exerts predictable and repeatable behavior that influenced the swimming capabilities of these living animals. Cephalopods with larger whorl expansion generally have shorter body chamber ratios (Trueman 1940; Saunders and Shapiro 1986; Klug 2001; Korn and Klug 2003; Klug and Korn 2004; Doguzhaeva and Mapes 2015; Peterman and Ritterbush 2022), lowering the mass distribution relative to the center of buoyancy, and increasing stability. The *Nautilus* model and theoretical oxycone in the current study experience quicker damping and more frequent oscillations compared to the serpenticone and sphaerocone with lower hydrostatic stabilities (Figs. 5 and 6; Supplementary Table S1). These experiments demonstrate that the stable morphotypes have considerably higher restoring moments acting to return the living animals to their static, equilibrium orientation. This condition would also reduce rocking during locomotion in response to jet thrust, and external forms of energy (e.g., current energy; wake produced by other organisms and water flowing over bathymetric features). Conversely, less stable morphotypes experience weaker restoration with fewer oscillations and generally slower movement (Fig. 7). While the separation between the hydrostatic centers can be incredibly low (∼0.6 mm; Supplementary Table S1), the corresponding stability was high enough to sufficiently return the cephalopods back to their preferred orientation. However, this low-stability condition would allow the living cephalopods to modify their orientations more easily with active jetting. This improved pitch maneuverability could make it easier to catch small prey items at different pitch angles. Additionally, lower stability could allow easier access to the benthos by tilting the aperture downward.

External shape also governs hydrodynamic restoration, although to a lesser extent than hydrostatics. During restoration, more inflated shapes incur more hydrodynamic drag, increasing damping. The *Nautilus* and sphaerocone models exemplify this behavior, experiencing higher damping despite having lower hydrostatic stability indices compared to the oxycone and serpenticone models, respectively (Fig. 5; Supplementary Table S1). Because external conch shape and rotational drag are factors involved in hydrodynamic restoration, perhaps the stunning array of ornamentation patterns of extinct ectocochleates (primarily the ammonoids; Wright et al. 1996) had some advantages/consequences for attenuating rocking. Heavy ornamentation patterns (coarse ribbing, nodes, tubercles, etc.) are more often expressed by less stable morphotypes (specifically, evolute conchs approaching the center of the morphospace Wright et al. 1996; Yacobucci 2004; Monnet, De Baets et al. 2015). While the current experiments only concern first-order conch coiling, the investigated properties could have been modulated by these second-order features (Chamberlain and Westermann 1976), including conch ornament, venter morphology (e.g., keels, carinae, and furrows) and apertural modifications (e.g., lappets, rostra, varices, etc.). Changes in scale and shape through ontogeny likely influenced the life habits of these living animals. Even planispirals experience considerable allometry (Korn 2012; De Baets et al. 2015) and must navigate changing physical properties throughout the lifespan of individual cephalopods. Additionally, the hydrodynamic consequences of disparate soft body morphologies and arm positioning could have modified these trends (e.g., extant squid using arms as control surfaces; Bartol et al. 2022). While the current models are equipped with conservative soft bodies, they align with the few exceptionally preserved ammonoid specimens available (Lehmann 1975, 1985; Klug et al. 2012, 2021; Klug and Lehmann 2015; Hoffmann, Morón-Alfonso et al. 2021; Cherns et al. 2022). Potential disparities can be explored in future studies as our understanding of soft body morphology is refined. Various combinations of these second-order factors can also be explored in future studies to better understand the relationships between conch form and function.

### Reevaluating cephalopod swimming capabilities and life habits

**Fig. 10:**
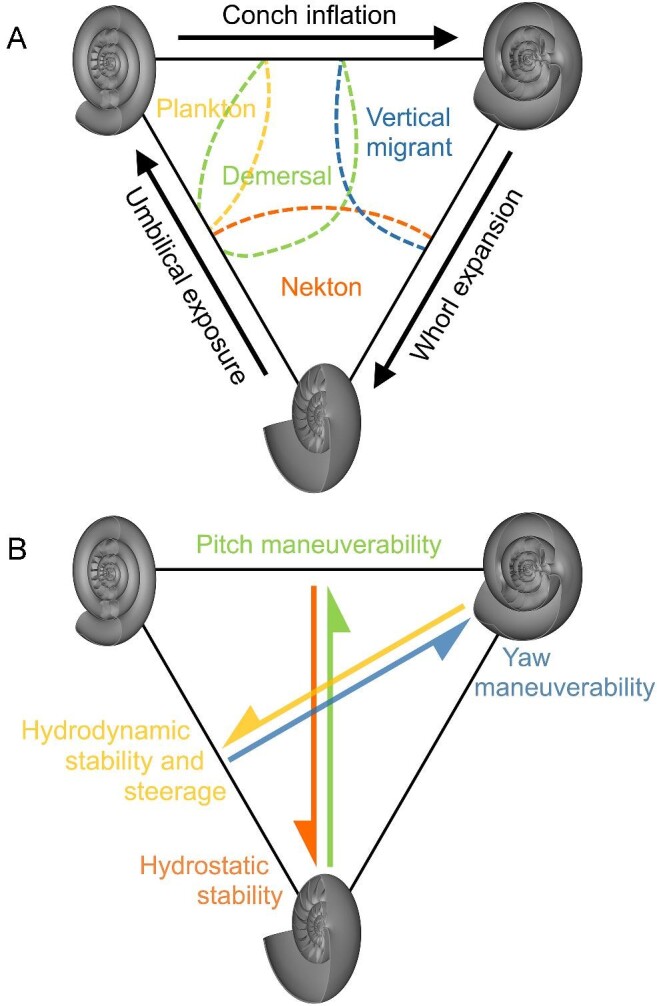
Reevaluation of swimming capabilities across the planispiral cephalopod morphospace. The Westermann morphospace (Ritterbush and Bottjer 2012) is represented as a ternary diagram with three endmembers (oxycones with high whorl expansion; serpenticones with high umbilical exposure; and sphaerocones with high conch inflation). **A** Historical interpretation of life habits across the morphospace (modified from Westermann 1996). Black arrows denote increasing conch parameters. **B** Proposed performance landscape with arrows indicating functional tradeoffs between (1) hydrostatic stability and pitch maneuverability and (2) hydrodynamic stability (coasting efficiency and steerage) and yaw maneuverability. Note that the top edge of the triangle represents morphologies with high pitch maneuverability while the bottom corner represents morphologies with high hydrostatic stability. The left edge of the triangle represents morphologies with high hydrodynamic stability, while the right corner represents morphologies with high yaw maneuverability.

Common interpretations of life habits (Fig. 10A) across the planispiral morphospace regard serpenticones and sphaerocones as hydrodynamically inferior compared to oxycones (Westermann 1996). Thus, cephalopods with these conch shapes have been speculated to assume planktic modes of life, as drifters or vertical migrants, respectively (Fig. 10A). However, strictly planktic interpretations for these entire morphogroups are unlikely. While no ectocochleate was likely to be as athletic or maneuverable as most fish or decabrachian cephalopods (Jacobs and Chamberlain 1996; Neil and Askew 2018), they would have been able to swim at comparable speeds (within an order of magnitude) compared to extant *Nautilus*, provided that they could produce rather conservative jet thrust (Hebdon et al. 2021; Hebdon, Ritterbush et al. 2022; Peterman and Ritterbush 2022; Ritterbush and Hebdon 2022). Swimming speed is probably not the best metric of performance for ectocochleates. Many heteromorphs (non-planispiral ammonoids) flourished during the Mesozoic despite their even less hydrodynamically sensible morphologies (Hoffmann, Slattery et al. 2021). These ammonoids displayed a wide range of unique hydrostatic properties (Okamoto 1996; Peterman, Ciampaglio et al. 2019; Peterman, Mikami et al. 2020; Peterman, Shell et al. 2020; Hoffmann, Slattery et al. 2021; Peterman et al. 2022) that may have contributed to their success occupying low-energy lifestyles. Like heteromorphs, planispirals assumed diverse ecological niches and iteratively evolved particular conch shapes throughout their long-lived evolutionary history (Bayer and McGhee 1984; Wright et al. 1996; Monnet et al. 2011; Monnet, Klug et al. 2015), suggesting adaptive benefits for disparate morphologies across the planispiral morphospace.

Regarding vertical migration potential, cephalopods with higher hydrostatic stability are better suited to moving in a wide array of directions (i.e., less sensitive to the thrust angle; Okamoto 1996; Klug and Korn 2004; Peterman, Mikami et al. 2020; Peterman, Shell et al. 2020; Peterman, Yacobucci et al. 2020). That is, morphotypes like oxycones, or others with short body chambers, are less sensitive to misalignment of their jetting direction with the hydrostatic centers (Fig. 1C). Under this condition, the stronger restoring moments demonstrated by the current experiments would have counteracted rocking. Extant nautilids frequently take part in vertical migration using active locomotion (Dunstan et al. 2011), and having relatively high hydrostatic stability (Supplementary Table S1) is key for this particular mode of life. Alternatively, morphotypes with low hydrostatic stability (like serpenticones and sphaerocones) would have been more sensitive to jetting in alignment with their hydrostatic centers for directional movement to occur. Depending on the propulsive potential of these living animals, multiple jet pulses may have been used to first alter orientation (pitch), then jet with the proper thrust angle. However, this mode of locomotion may be less efficient because it would require multiple steps, while fighting hydrodynamic restoration during the mantle cavity recovery phase. It is more likely that morphologies with more rapid whorl expansion had improved vertical movement potential (rather than sphaerocones; see Westermann 1996; Ritterbush and Bottjer 2012), although this form of locomotion would not be the only capability of these living animals. For example, oxycones would have improved directional movement potential and coasting efficiency in a variety of directions, improving their general motility. While many disparate cephalopods occupied different depths through ontogeny (indicated by isotopic analyses; Lukeneder 2015), diurnal migration would likely be time-averaged (Linzmeier 2019) and generally unresolvable. Therefore, hydrostatic analyses serve as an alternative proxy for short term vertical movement capabilities by providing some physical constraints on this behavior.

Rather than binning morphogroups into particular life habits, perhaps evaluating the physical constraints of these morphologies would have more value for determining the ecological roles of ectocochleates (Fig. 10B), especially when combined with isotopic analyses (Lukeneder 2015; Sessa et al. 2015), biotic associations (Tsujita and Westermann 1998), lithofacies associations (Batt 1989; Kawabe 2003), and the paleobiology of individual taxa. It is unlikely that any planispiral conch shape would enforce a strictly planktic life habit (aside from the planktic life stages of hatchling ammonoids; De Baets et al. 2015). Oxycones are well streamlined and incur lower hydrodynamic drag (at larger sizes and/or speeds—Reynolds numbers; Jacobs 1992; Jacobs and Chamberlain 1996; Hebdon et al. 2020, 2021; Hebdon, Ritterbush et al. 2022; Peterman and Ritterbush 2022; Ritterbush and Hebdon 2022). Cephalopods approaching this endmember also have superior hydrodynamic stability (Bayer 1982; Peterman and Ritterbush 2022) and hydrostatic stability (Peterman and Ritterbush 2022), improving coasting efficiency (Hebdon, Ritterbush et al. 2022), directional control (Bayer 1982; Jacobs and Chamberlain 1996), and minimizing rocking during locomotion. Oxycones indeed seem well suited to life in higher energy environments, which is supported by fossil occurrences, lithofacies analysis, and isotopic analyses (Kennedy and Cobban 1976; Batt 1989; Jacobs et al. 1994; Kawabe 2003; Monnet, De Baets et al. 2015; but note that opposite trends are reported as well, e.g., Wilmsen and Mosavinia 2011). However, these benefits come at the expense of maneuverability. High hydrodynamic stability costs more energy to change yaw (Peterman and Ritterbush, 2022; Fig. 1), and high hydrostatic stability impedes changes in pitch. The complex topology of serpenticone flanks does not produce enough drag to reduce these animals to planktic life habits (Hebdon, Ritterbush et al. 2022; Peterman and Ritterbush 2022). Low hydrostatic stability in this morphotype would improve pitch maneuverability, but make these living animals more sensitive to jetting through the hydrostatic centers to produce efficient translation and minimize rocking (Peterman and Ritterbush 2022). Their laterally compressed conchs (similar to oxycones) would provide high hydrodynamic stability, improving coasting efficiency, but reducing yaw maneuverability (Peterman and Ritterbush 2022). Sphaerocones seem to be optimized for maneuverability about the vertical and horizontal axes. While sphaerocones of the same size as other morphotypes (by volume) could not accelerate as fast or reach the same maximum velocities (Peterman and Ritterbush 2022), they were highly maneuverable in any direction. For fishes, predator evasion potential can be improved by higher swimming velocities and acceleration, or higher maneuverability (Howland 1974; Webb 1984; Fish 2002; Weihs 2002). Higher maneuverability for certain ectocochleates may have decreased predation risk for slower predators, or denied soft body access with quicker turns (Peterman and Ritterbush 2022; Peterman et al. 2022). However, it seems improved maneuverability may be most beneficial for feeding on small prey items in lower energy environments. That is, these cephalopods would have reduced energy expenditure and self-generated wake during rotation while having 360° access to closely surrounding prey (Peterman and Ritterbush 2022; Peterman et al. 2022). In addition to these physical properties, other factors are likely involved in selection (other functional/morphogenetic factors, Kröger 2005; Tendler et al. 2015; Parent et al. 2020; Peterman et al. 2021; Hebdon, Polly et al. 2022; Weber et al. 2022; competition, Ritterbush 2016). While the endmembers of the planispiral morphospace (Ritterbush and Bottjer 2012) represent extreme cases, the majority of cephalopods occupied spaces in between. The proposed functional characteristics across our newly introduced performance landscape (Fig. 10B) can be used to assess relative functional constraints for cephalopods that may represent generalists or intermediates between particular stability–maneuverability endmembers.

### Using coiling parameters as proxies for biomechanical constraints

Our proposed performance landscape of physical tradeoffs (Fig. 10B) can be used to investigate the ecological roles between clades, and how certain groups responded to environmental perturbations (extinction and recovery). The current experiments demonstrate whorl expansion can be used as a proxy for hydrostatic stability (with lower expansion indicating higher pitch maneuverability). Previous studies (Bayer 1982; Hebdon, Ritterbush et al. 2022; Peterman and Ritterbush 2022) reveal that conch compression is a proxy for hydrodynamic stability and coasting efficiency (with more inflated conchs having better yaw maneuverability). Investigating the morphological disparity of Paleozoic ammonoid orders reveals several distinct biomechanical constraints. While Goniatitida occupies a wide range of the morphospace (Fig. 8B), this group more frequently expresses inflated conchs with low whorl expansion. These forms would have emphasized both pitch and yaw maneuverability, making them well suited to low-energy lifestyles while improving the capture of small prey items. The clymeniids (Fig. 8C) and early ceratitids (Fig. 8E) occupy similar regions of the morphospace (Supplementary Tables S9 and S10) and more frequently express serpenticonic forms, emphasizing better hydrodynamic stability and pitch maneuverability. Prolecanitids more frequently express oxyconic shapes (Fig. 8D) which emphasize both hydrostatic and hydrodynamic stability. These ammonoids are better suited to cruising longer on a single jet and have improved directional motility (horizontal and vertical). Furthermore, they are more resistant to external or self-generated perturbations during locomotion. While not strictly confined to any particular environment, these shapes would have been able to tolerate shallower/proximal settings with more ambient turbulence and wave action. Lastly, Agoniatitida generally has intermediate whorl expansion and conch compression, perhaps reflecting generalist swimming capabilities. These morphologies near the center of the morphospace may not frequently optimize stability or maneuverability, but have intermediate performances in each category. In addition to selection for various functional properties, these groups exhibit a strong degree of phylogenetic control on morphology. That is, most of the examined groups span tens of millions of years with little change in conch parameters. Even after extinction events (e.g., the Devonian–Carboniferous), goniatitids express more laterally compressed conchs, but gradually expand into the previously occupied portion of the morphospace (Supplementary Fig. S7). Conversely, in the later portion of their ranges, agoniatitids seem be to be selected for more compressed conchs, while prolecanitids (some species within Daraelitidae) experiment with inflated conchs (Supplementary Fig. S7). These deviations from the center of their respective distributions, and iterative appearance of certain morphotypes, suggest that disparate shapes offered adaptive value for particular life habits and swimming performances. Representing the examined species with a single set of coiling parameters at adulthood also complicates our understanding of the life habits of these animals because they experience considerable allometry (Korn and Klug 2001). However, the general biomechanical relationships hold true for individual life stages of these animals, despite earlier stages being underrepresented in the morphospace analyses. Nonetheless, this test case demonstrates that the examined groups experience distinctive physical constraints imposed by their disparate conch morphologies, which likely reflects a similar degree of functional diversity. The diverse lifestyles permitted by these morphologies and their rapid evolution during the Paleozoic illuminate the many selective opportunities presented by a form of aquatic locomotion rather unique to ectocochleates.

Ammonoid diversity and disparity generally decline during every survived mass extinction (Smith et al. 2014; Brayard and Bucher 2015; Landman et al. 2015; Longridge and Smith 2015). After these events, ammonoids generally recover until they occupy the previous regions of their morphospace. The Triassic–Jurassic extinction is an excellent test case for this behavior because ammonoids are characterized mostly by serpenticones during the aftermath of this event (Smith et al. 2014; Fig. 9B). During the post-extinction and recovery intervals (Fig. 9C, D), ammonoids once again experiment with varying degrees of conch inflation and whorl expansion. These differences in morphology suggest that Triassic–Jurassic extinction (and Devonian–Carboniferous extinction for goniatitids) selected for laterally compressed conchs. However, planispiral cephalopods more frequently express some degree of shell compression (Raup 1967; whorl height to width ratio >1), suggesting this condition may be the “status quo.” In other words, the center of the morphospace is not the most frequently occupied portion. It is unclear whether the abundance of serpenticones reflects selection for particular swimming capabilities. However, these shapes would have imposed constraints on life habits and ecological roles. Other factors could have been involved in selection, involving other functions (soft body retraction; Kröger 2002), or morphogenetic factors (shell economy; Tendler et al. 2015). Combinations of these functional, morphogenetic, and phylogenetic factors can be explored together with emerging tools like joint fitness landscapes (Hebdon, Polly et al. 2022), especially when single factors do not adequately predict the range of morphologies preserved in the fossil record. Furthermore, as more comprehensive datasets are openly shared, these analyses can be used to explore the dynamics of selection, extinction, and evolution more broadly.

### Importance of hydrostatics and mass distribution in aquatic biomimetics and bioinspiration

The new techniques developed to replicate the mass distributions of living animals can be applied to other biomechanical models or bioinspired technologies (e.g., remotely operated underwater vehicles). Differing material properties (i.e., masses and densities) between physical models and the organisms they represent, create barriers to constructing lifelike biomechanical models and aquatic robots. Total model mass must be managed to account for proper buoyancy. The distribution of mass (depending on the 3D placement of each material of which it is composed) affects orientation, stability, and the total moment of inertia (i.e., rotational kinematics). Each of these properties must be replicated to completely model the full, 3D movement of a submerged object in any direction. However, their relative importance differs by test case and form of movement.

### Diverse functional opportunities across the morphospace

While the detailed dynamics of selection, extinction, and evolution are obscure for planispiral cephalopods, the current experiments demonstrate that conch morphology imposes different physical advantages and consequences across their morphospace. Different combinations of stability–maneuverability tradeoffs (Fig. 10) presented a host of opportunities for the swimming performances and function of these living animals. It is unlikely that these are the only factors involved in selection, however, the proposed scheme can be used to better assess the constraints on the life habits of these animals and their ecological roles.

The high hydrostatic stability, hydrodynamic stability, and lower drag experienced by oxycones (specifically at higher Reynolds numbers; Jacobs 1992; Hebdon et al. 2021) intuitively suggest that this shape confers the most advantages for active swimming. However, these abilities sacrifice performance in terms of maneuverability. These tradeoffs reinforce that there is no single optimum morphology in aquatic biomechanics (Webb 1984, 2002; Jacobs 1992; Weihs 2002), especially among rigid-bodied animals like ectocochleates. The iterative appearance of disparate conch morphologies that deviate from streamlined forms, and the examined biomechanical tradeoffs, demonstrate that no ectocochleate is universally adapted for high performance in every category. The distinct physical properties of these cephalopods depend upon conch geometry; adding value to their fossils as tools to study evolutionary biomechanics and life's responses to global change.

## Supplementary Material

obac048_Supplemental_File

## Data Availability

All virtual hydrostatic models are stored on an online database (see Dataset 1 of the Supplementary Information; DOI: 10.5281/zenodo.6316035).
