# Supplementary material for: Stability–Maneuverability Tradeoffs Provided Diverse Functional Opportunities to Shelled Cephalopods
Source: Integr Org Biol. 2022 Nov 9;4(1):obac048. doi: 10.1093/iob/obac048 (PMC9743176; doi:10.1093/iob/obac048)
Supplement: obac048_Supplemental_File [file obac048_supplemental_file.pdf]

Supplementary materials for

Stability-maneuverability tradeoffs provided diverse functional opportunities to shelled cephalopods

David J. Peterman and Kathleen A. Ritterbush

*Corresponding author email: David.Peterman@utah.edu

This PDF file includes

Figures S1 to S7

Tables S1 to S11

Legend for Dataset S1

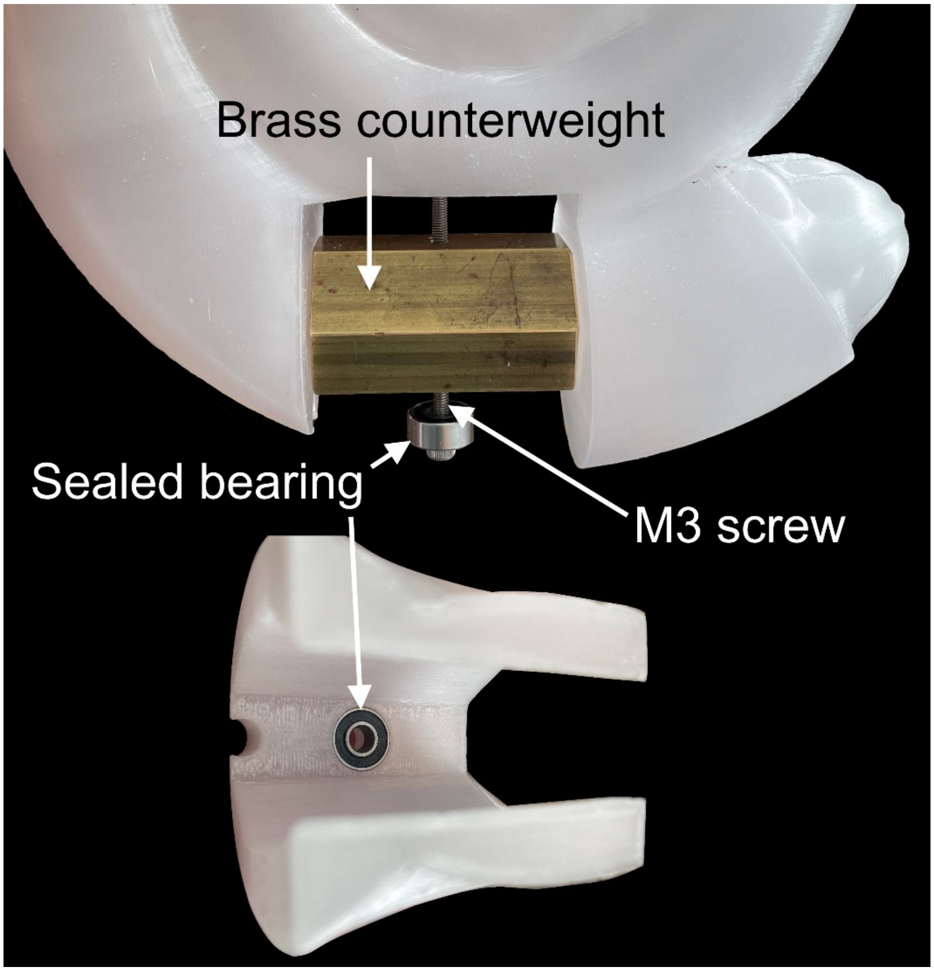

Figure S1: Assembly of 3D-printed, physical model showing counterweight design. The brass counterweight can move up and down an M3 screw to adjust the total center of mass, using a computed number of screw turns. A sealed bearing permits the screw to rotate while keeping this cavity water-tight, which secondarily functions as a buoyancy adjustment chamber.

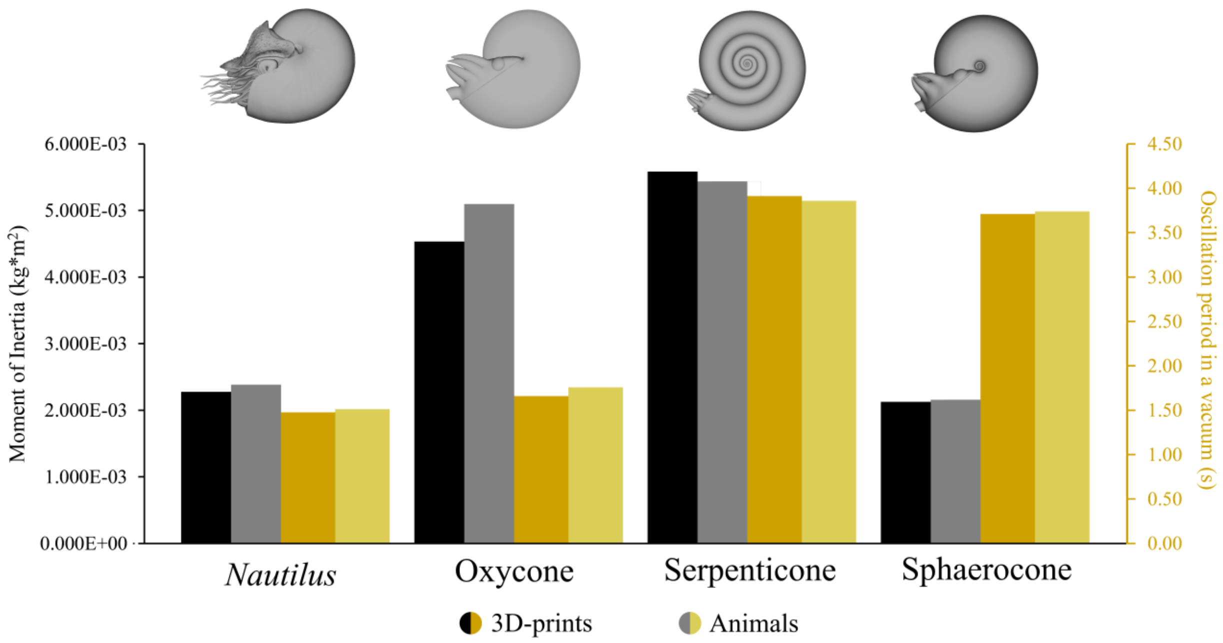

Figure S2: Moments of inertia (black and grey; left axis) and oscillation period in a vacuum (dark and light gold; right axis) for each examined morphology. Dark colors denote the physical, 3D-printed models (3D-prints), and light colors denote the virtual hydrostatic model representing the living animals (Animals). When volume is held constant, more discoid shapes generally have higher moments of inertia because mass is distributed farther from the rotational axis. Oscillation period computed in a vacuum depends upon hydrostatic stability (distance between the centers of buoyancy and mass), and moment of inertia (Period = $2\pi*(I/mgd)^{0.5}$; where I = moment of inertia, m = mass, g = gravitational acceleration, d = distance between the hydrostatic centers). Differences in each value betwween the physical and virtual models are reported in Table S2.

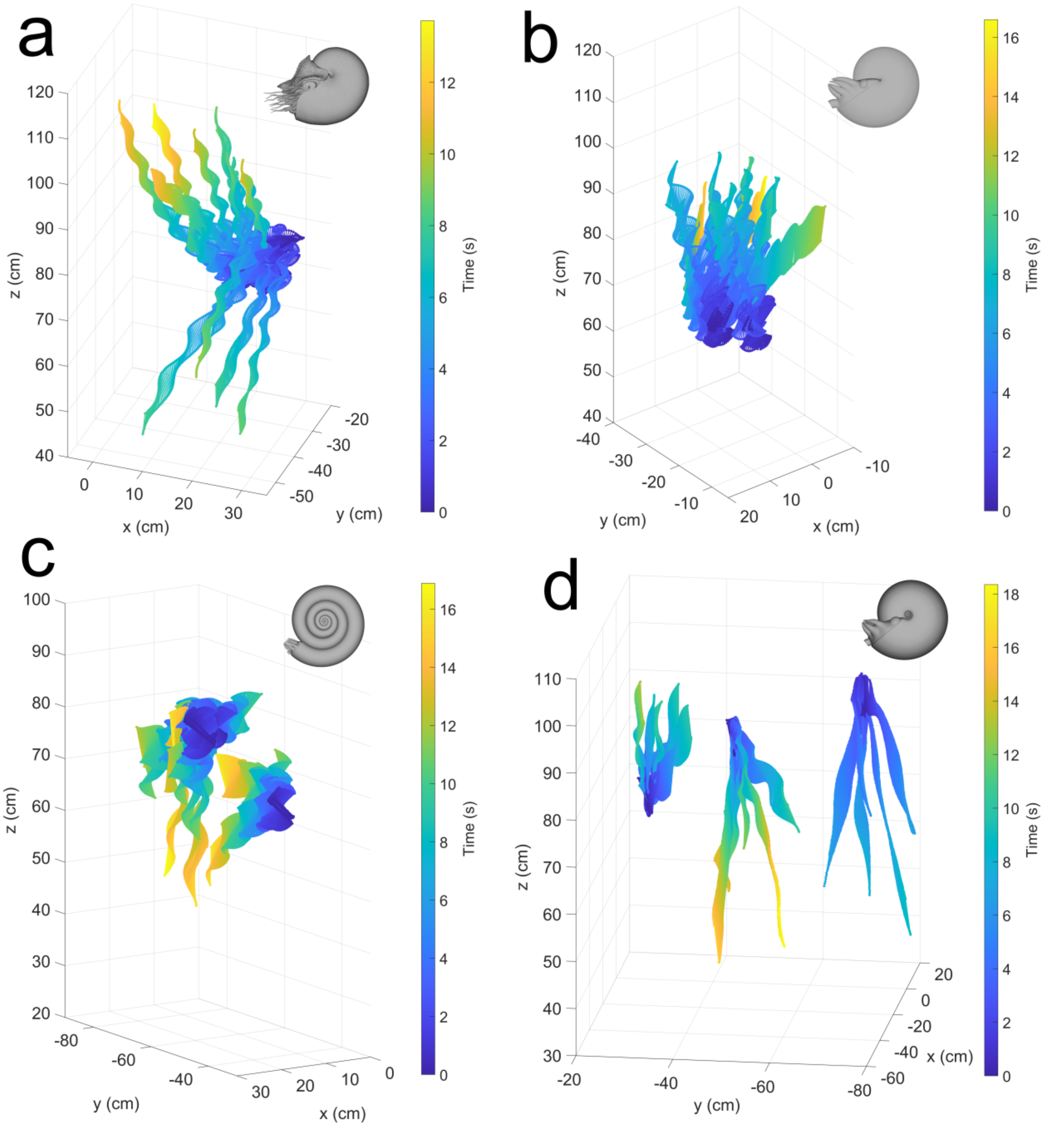

Figure S3: 3D positions of the upper and lower tracking points (each connected by a line for each timestep). Each model was rotated ~55 degrees so that the initial direction of rotation was aperture backwards. Rocking was monitored for 15 trials each: a) *Nautilus*, b) oxycone, c) serpenticone, and d) sphaerocone. Colors denote the time since the model was released. Model rocking was monitored until the tracking points became obsured (too much yaw rotation), or the model approached boundaries (i.e., water surface or pool bottom).

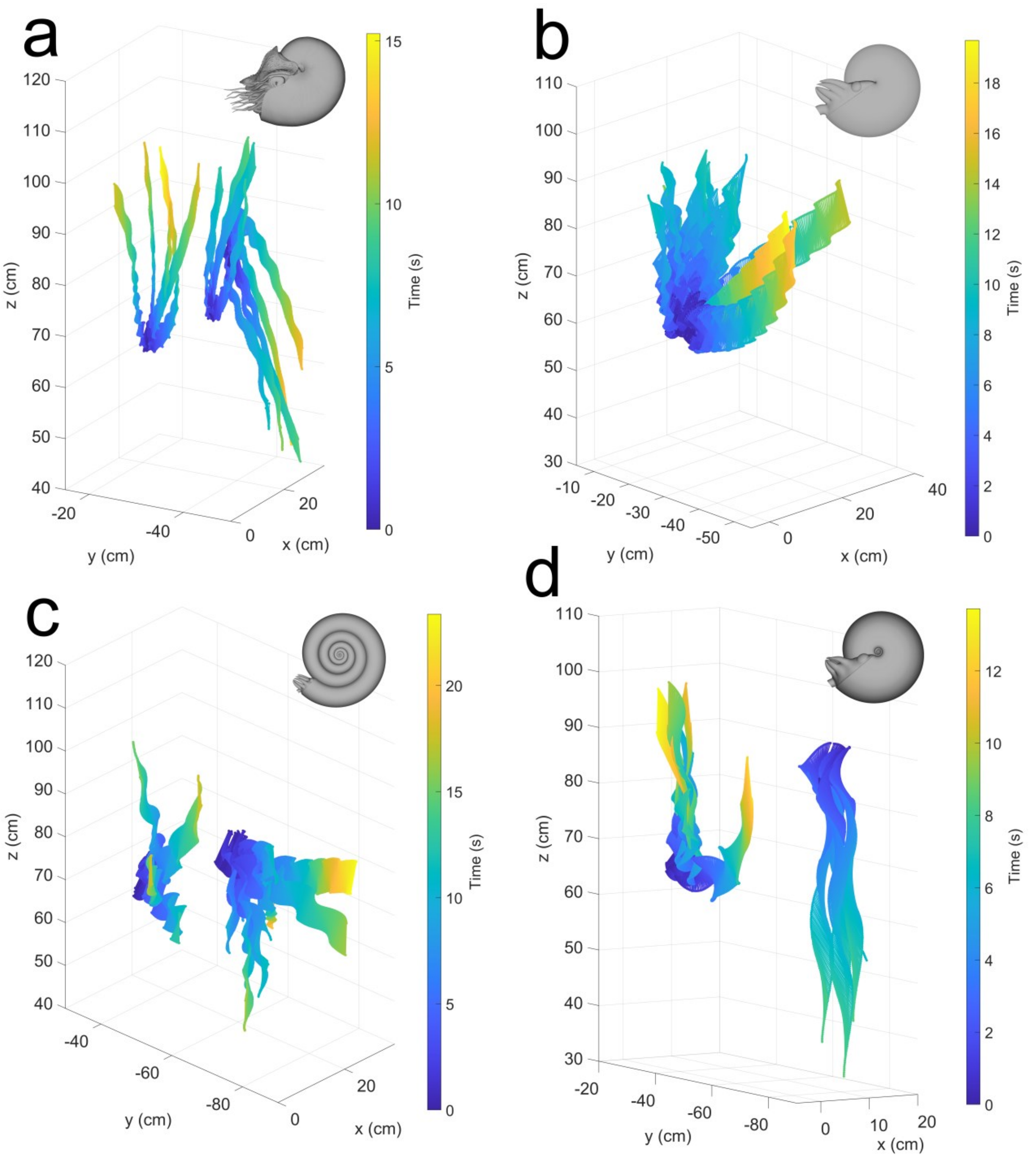

Figure S4: 3D positions of the upper and lower tracking points (each connected by a line for each timestep). Each model was rotated ~55 degrees so that the initial direction of rotation was aperture forwards. Rocking was monitored for 15 trials each: a) *Nautilus*, b) oxycone, c) serpenticone, and d) sphaerocone. Colors denote the time since the model was released. Model rocking was monitored until the tracking points became obsured (too much yaw rotation), or the model approached boundaries (i.e., water surface or pool bottom).

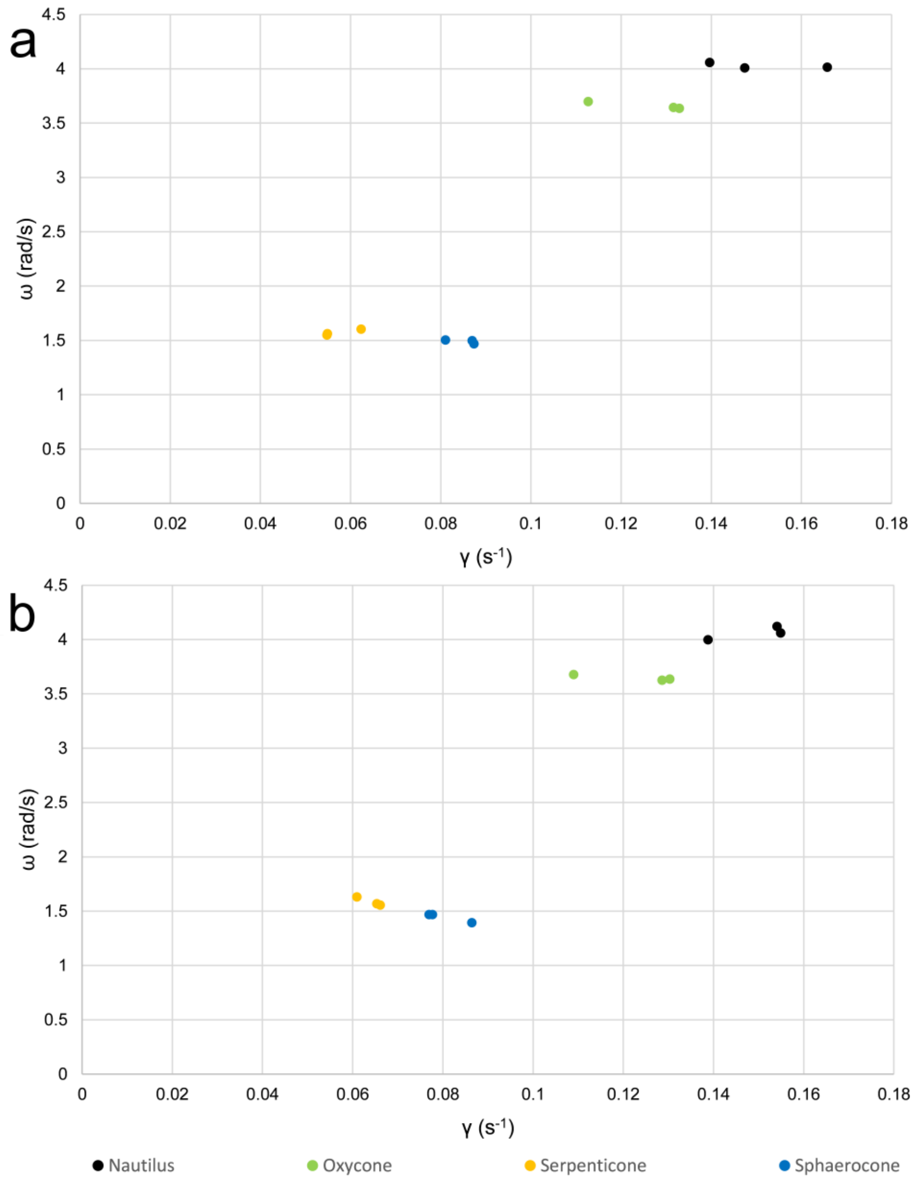

Figure S5: Comparison of damping coefficients (γ) and angular frequencies (ω). The adjustable counterweights were reset between each five trials, producing a total of 15 trials for each model. Each point represents a grouping of each 5 trials where the counterweights had yet to be reset. a) aperture-backwards release, b) aperture-forwards release.

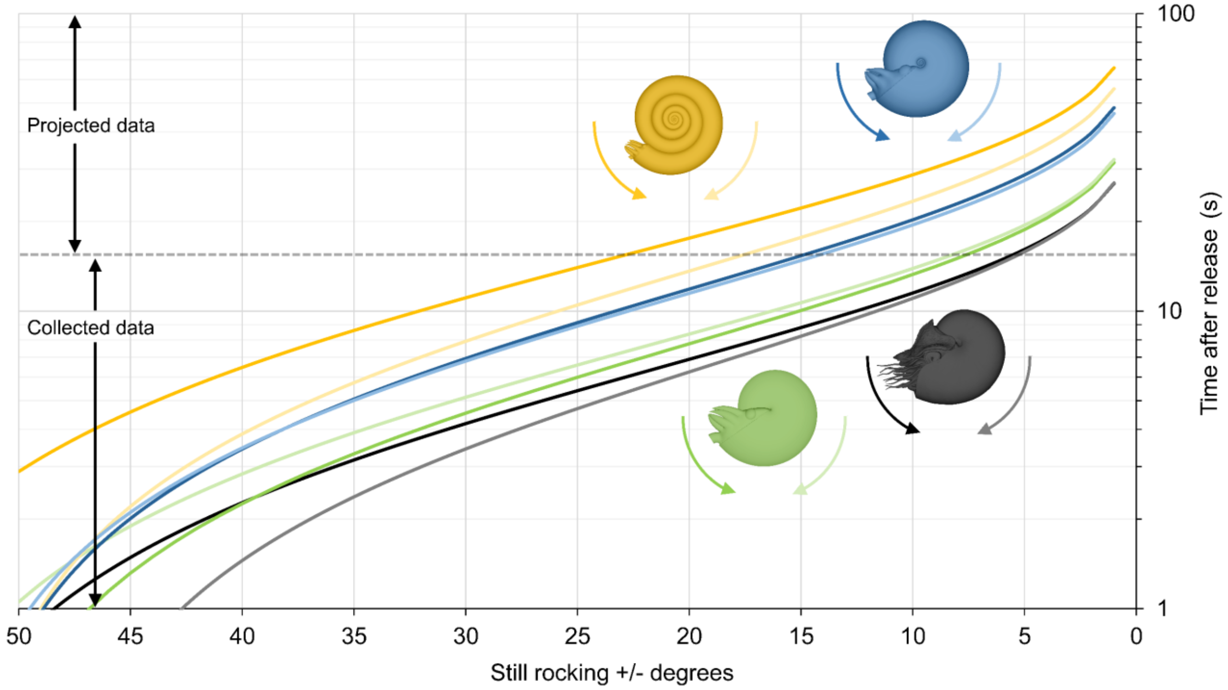

Figure S6: Model oscillation (rocking) after being released from ~50-60 degrees from their static orientations. Times were computed from rearranging Equation 7 and only considering the exponential decay of the function (setting angular frequency, ω, to zero). Note that the y axis is in a logarithmic scale. Hydrostatically unstable morphotypes (serpenticone and sphaerocone) take much longer to reach smaller rocking angles compared to hydrostatically stable morphotypes (*Nautilus* and oxycone).

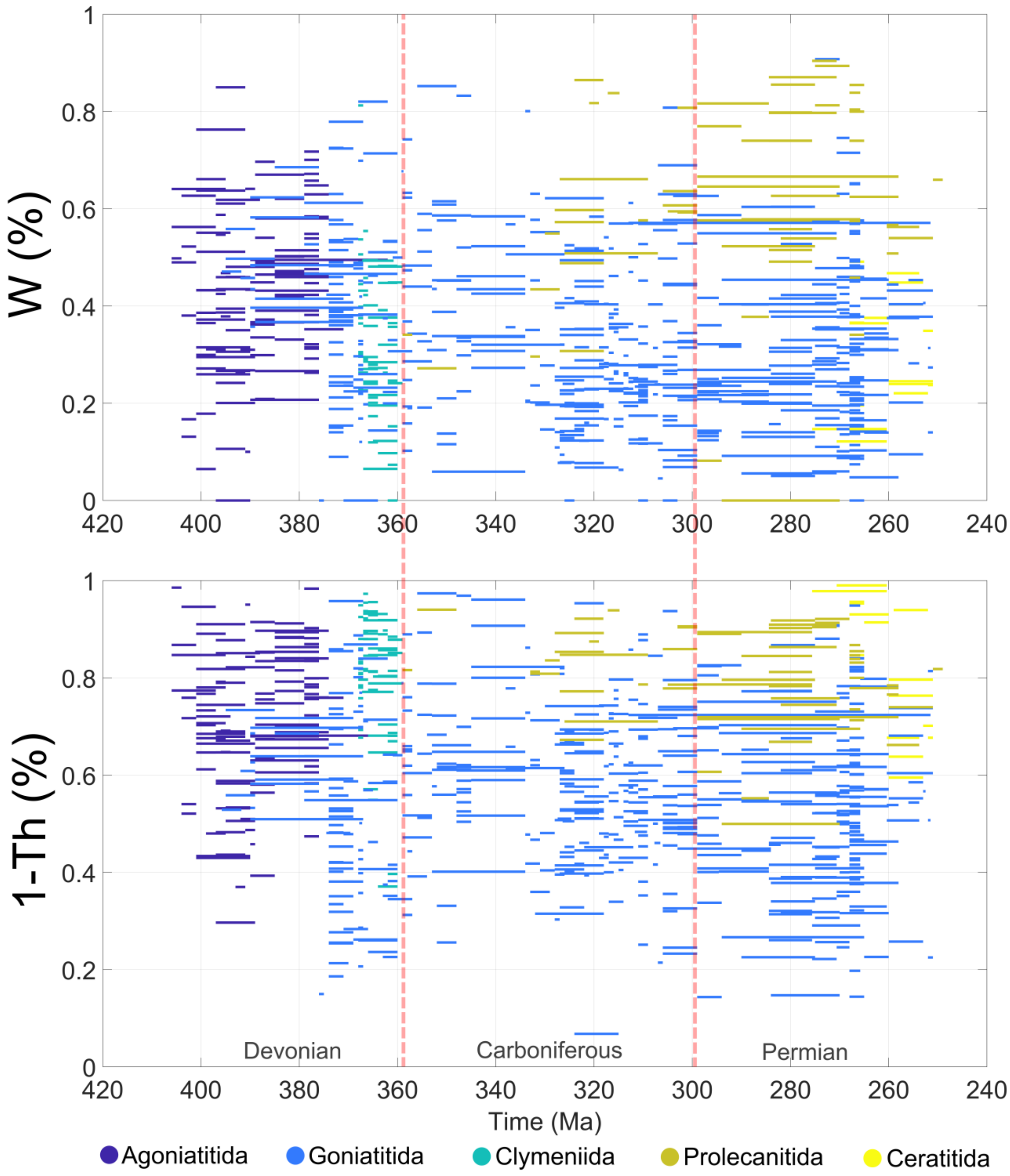

Figure S7: Whorl expansion (W) and compression ratio (1-Th) plotted through time for five Paleozoic ammonoid orders. Conch measurements, FADs, and LADs from Whalen et al. (2020). Red dashed lines correspond to boundaries between geologic periods.

Table S1: Hydrostatic stability index (St) computed from virtual hydrostatic models (Peterman et al., 2018; Peterman and Ritterbush, 2022). All related hydrostatic parameters of the current models were computed after scaling them to nearly identical volumes and masses (Table S1). Note that computed screw turns were rounded, since exact values could not be reliably imparted in the models.

Model	S_t	BM (mm)	Counterweight distance moved (mm)	Screw turns (revolutions)	Screw turns rounded (revolutions)
Nautilus	0.043	4.278	18.613	37.225	37.2
**Oxycone**	0.068	6.765	29.329	58.640	58.6
**Serpenticone**	0.015	1.492	6.491	12.983	13.0
**Sphaerocone**	0.006	0.597	2.594	5.187	5.2

Table S2: Moments of inertia computed for each component of the 3D-printed model (3D Print) and the virtual hydrostatic model representing the living animal (animal). Because the 3D-printed models are constructed with materials of differing physical properties from the living animals (densities, volumes, and local mass distributions), the total moments of inertia between each model were compared. Additionally, the periods of oscillation (in a vacuum) were computed from moments of inertia and the distances between the centers of buoyancy and mass for the 3D-printed models and virtual models. The percent differences in moments of inertia and oscillation periods between the 3D-printed models and virtual models suggest that deviations from this property do not considerably alter rotational kinematics.

	Nautilus		**Oxycone**		**Serpenticone**		**Sphaerocone**	
**Material**	**Model**	**Animal**	**Model**	**Animal**	**Model**	**Animal**	**Model**	**Animal**
**I PETG (kg*m^2)**	1.871E-03		3.987E-03		3.935E-03		1.785E-03	
**I Brass counterweight (kg*m^2)**	2.868E-04		3.751E-04		1.177E-03		2.189E-04	
**I Gallium counterweight (kg*m^2)**	9.542E-05		1.338E-04		3.976E-04		9.884E-05	
**I Screw (kg*m^2)**	4.601E-06	NA	8.258E-06	NA	1.461E-05	NA	4.729E-06	NA
**I Bearing (kg*m^2)**	9.295E-06		1.450E-05		2.215E-05		9.492E-06	
**I Self-healing rubber (kg*m^2)**	9.875E-07		1.878E-06		2.258E-06		9.202E-07	
**I Liquid (kg*m^2)**	7.263E-06		1.223E-05		3.654E-05		7.425E-06	
**I Soft body (kg*m^2)**	NA	1.933E-03	NA	3.408E-03	NA	4.156E-03	NA	1.815E-03
**I Shell (kg*m^2)**		4.527E-04		1.689E-03		1.281E-03		3.434E-04
**Total I (3D Print) (kg*m^2)**	2.275E-03	NA	4.532E-03	NA	5.585E-03	NA	2.125E-03	NA
**Total I (animal) (kg*m^2)**	NA	2.385E-03	NA	5.097E-03	NA	5.438E-03	NA	2.159E-03
**% Difference in I**	**4.62**		**11.07**		**-2.71**		**1.54**	
**Oscillation period in a vacuum (3D Print) (s)**	1.47	NA	1.66	NA	3.91	NA	3.71	NA
**Oscillation period in a vacuum (animal) (s)**	NA	1.51	NA	1.76	NA	3.86	NA	3.74
**% Difference in period**	**2.34**		**5.70**		**-1.35**		**0.77**	

Table S3: Comparison of variables fit to Equation 7 for each set of five trials for each model. Counterweights were reset between each five trials. Starting angle = θ_0, damping coefficient = γ, and angular frequency = ω.

Model	Direction	Trials	θ_0 (degrees)	γ (s_{-1})	ω (rad/s)
Nautilus	Backwards	1-5	57.76	0.1657	4.014
Nautilus	Backwards	6-10	55.61	0.1474	4.008
Nautilus	Backwards	11-15	53.21	0.1396	4.058
Nautilus	Backwards	All	56.25	0.1502	4.024
Nautilus	Forwards	1-5	-50.24	0.1549	4.061
Nautilus	Forwards	6-10	-52.28	0.1388	3.999
Nautilus	Forwards	11-15	-52.05	0.1541	4.121
Nautilus	Forwards	All	-49.33	0.1445	4.062
Oxycone	Backwards	1-5	53.67	0.1316	3.644
Oxycone	Backwards	6-10	51.83	0.1127	3.699
Oxycone	Backwards	11-15	55	0.1329	3.635
Oxycone	Backwards	All	53.11	0.1258	3.654
Oxycone	Forwards	1-5	-58	0.1303	3.637
Oxycone	Forwards	6-10	-53.8	0.109	3.678
Oxycone	Forwards	11-15	-58	0.1286	3.625
Oxycone	Forwards	All	-57.05	0.1253	3.652
Serpenticone	Backwards	1-5	58.47	0.05484	1.563
Serpenticone	Backwards	6-10	60.87	0.06229	1.603
Serpenticone	Backwards	11-15	58.22	0.05475	1.55
Serpenticone	Backwards	All	59.87	0.06234	1.562
Serpenticone	Forwards	1-5	-51.73	0.06536	1.569
Serpenticone	Forwards	6-10	-49.89	0.06096	1.63
Serpenticone	Forwards	11-15	-52.42	0.06609	1.556
Serpenticone	Forwards	All	-52.62	0.07096	1.591
Sphaerocone	Backwards	1-5	53.51	0.08692	1.497
Sphaerocone	Backwards	6-10	54.55	0.08103	1.504
Sphaerocone	Backwards	11-15	57.55	0.08732	1.469
Sphaerocone	Backwards	All	53.09	0.08233	1.493
Sphaerocone	Forwards	1-5	-52.34	0.08642	1.393
Sphaerocone	Forwards	6-10	-53.51	0.07695	1.468
Sphaerocone	Forwards	11-15	-54.01	0.07771	1.468
Sphaerocone	Forwards	All	-54	0.08655	1.447

Table S4: Starting point (θ_0), damping coefficient (γ), angular frequency (ω) for each model and each experimented release direction (starting backwards or forwards). These variables were fit to Equation 7 using the curve fitting toolbox in MATLAB. Upper and lower 95% confidence intervals (CI), and R2 values were computed for each variable.

Model	Release direction	θ_0 (degrees)	θ_0 lower CI	θ_0 upper CI	γ (s^{-1})	γ lower CI	γ upper CI	ω (rad/s)	ω lower CI	ω upper CI	R^2
Nautilus	Backwards	56.25	55.56	56.95	0.1502	0.1467	0.1538	4.024	4.022	4.026	0.9468
Nautilus	Forwards	-49.33	-49.97	-48.70	0.1445	0.1411	0.1479	4.062	4.06	4.064	0.9323
Oxycone	Backwards	53.11	52.36	53.85	0.1258	0.1222	0.1294	3.654	3.652	3.657	0.9302
Oxycone	Forwards	-57.05	-57.80	-56.30	0.1253	0.1237	0.1268	3.652	3.651	3.654	0.9569
Serpenticone	Backwards	59.87	59.02	60.73	0.06234	0.05996	0.06472	1.562	1.561	1.564	0.9114
Serpenticone	Forwards	-52.62	-53.31	-51.93	0.07096	0.06884	0.07308	1.591	1.589	1.592	0.9125
Sphaerocone	Backwards	53.09	52.33	53.86	0.08233	0.0794	0.08525	1.493	1.491	1.495	0.9198
Sphaerocone	Forwards	-54.00	-54.08	-53.93	0.08655	0.08454	0.08855	1.447	1.445	1.449	0.9461

Table S5: Densities, volumes, and masses for each model component for each model. Note that the screw density for the oxycone is different than all others because a different material was used (4.956 g/cm^3). Minor differences in reported mass/volume values and reported densities are due to rounding.

		Nautilus		Oxycone		Serpenticone		Sphaerocone	
**Model component**	**Density (g/cm^3)**	**Volume (cm^3)**	**Mass (g)**	**Volume (cm^3)**	**Mass (g)**	**Volume (cm^3)**	**Mass (g)**	**Volume (cm^3)**	**Mass (g)**
**PETG**	1.264	568.000	717.952	573.971	725.500	550.800	696.211	568.000	717.952
**Brass counterweight**	8.529	26.540	226.365	26.642	227.240	26.545	226.410	26.577	226.680
**Gallium counterweight**	5.900	4.335	25.574	2.349	13.858	8.471	49.979	4.335	25.574
**M3 Screw**	5.096	0.646	3.291	0.904	4.480	0.646	3.291	0.646	3.291
**Bearing**	5.336	0.488	2.602	0.488	2.602	0.488	2.602	0.488	2.602
**Self-healing rubber**	1.568	0.181	0.284	0.158	0.247	0.181	0.284	0.181	0.284
**Liquid**	1.000	2.387	2.388	2.387	2.388	4.980	4.981	2.387	2.388
**Total**	~1.0002	984.686	984.883	984.684	984.881	984.688	984.885	984.687	984.884

Table S6: Local centers of mass for each model component, centers of buoyancy, and total centers of mass for each model. Note that the computed total center of mass imparts a zero-stability condition (i.e., occupies the same location as the center of buoyancy). The counterweight chamber was designed to allow enough movement to hydrostatically invert the model, and to impart the proper stability with the specified number of screw turns (Equation 5).

	Nautilus			**Oxycone**			**Serpenticone**			**Sphaerocone**		
**Model component**	**x**	**y**	**z**	**x**	**y**	**z**	**x**	**y**	**z**	**x**	**y**	**z**
**PETG**	0.000	0.010	3.731	0.000	0.008	2.713	0.000	0.010	15.996	0.000	0.009	6.107
**Brass counterweight**	0.000	0.000	-16.594	0.000	0.000	-12.000	0.000	-0.001	-64.645	0.000	0.000	-24.480
**Gallium counterweight**	0.000	0.000	58.609	0.000	0.000	94.750	0.000	0.000	88.123	0.000	0.000	61.522
**M3 Screw**	0.000	0.000	-32.579	0.001	-0.001	-33.519	0.000	0.000	-63.722	0.000	0.000	-31.389
**Bearing**	0.000	0.000	-61.795	0.000	0.000	-77.914	0.000	0.000	-92.937	0.000	0.000	-60.605
**Self-healing rubber**	0.000	-24.129	-61.045	0.000	-22.333	-90.489	0.000	-23.250	-89.864	0.000	-22.920	-57.172
**Liquid**	0.001	-0.074	-55.442	0.001	-0.027	-73.447	0.000	-0.042	-85.187	0.000	-0.074	-54.251
**Center of buoyancy**	0.000	0.000	0.000	0.000	0.000	0.000	0.000	0.000	0.000	0.000	0.000	0.000
**Center of mass**	0.000	0.000	0.000	0.000	0.000	0.000	0.000	0.000	0.000	0.000	0.000	0.000

Table S7: Computed time to reach certain rocking thresholds (t_{10} = ± 10°; t_5 = ± 5°). Upper and lower 95% confidence intervals for $\boldsymbol{\theta_0}$ and $\boldsymbol{\gamma}$ were used to address computed variations. Times were computed from rearranging Equation 7 and only considering the exponential decay of the function (setting angular frequency, $\boldsymbol{\omega}$, to zero).

**Model**	**Direction**	**t_{10} (s)**	**t_{10} (s) lower CI**	**t_{10} (s) upper CI**	**t_5 (s)**	**t_5 (s) lower CI**	**t_5 (s) upper CI**
Nautilus	Backwards	11.50	11.69	11.31	16.11	16.41	15.82
Nautilus	Forwards	11.04	11.40	10.70	15.84	16.31	15.39
Oxycone	Backwards	13.27	13.55	13.01	18.78	19.22	18.37
Oxycone	Forwards	13.90	14.18	13.63	19.43	19.79	19.10
Serpenticone	Backwards	28.71	29.61	27.87	39.83	41.17	38.58
Serpenticone	Forwards	23.40	24.31	22.54	33.17	34.38	32.03
Sphaerocone	Backwards	20.28	20.84	19.75	28.70	29.57	27.88
Sphaerocone	Forwards	19.48	19.96	19.03	27.49	28.16	26.86

Table S8: Standard deviations (mm) of reconstructed tracking point distances for each recorded video used in 3D motion tracking.

Model	**Tracking point distance (mm)**	**Video 1**	**Video 2**	**Video 3**	**Video 4**	**Video 5**
Nautilus	43.5	1.11	0.89	0.78	NA	NA
Oxycone	89.0	1.10	0.87	0.77	0.83	1.10
Serpenticone	76.0	0.86	1.90	0.98	NA	NA
Sphaerocone	74.0	1.08	0.69	0.56	0.81	NA

Table S9: Whorl expansion (W; used as a proxy for hydrostatic stability), 1-Th (compression ratio, used as a proxy for hydrodynamic stability), and umbilical exposure (U) for five Paleozoic ammonoid orders. Each coiling parameter (W, Th, U) represent percentages on a ternary diagram (see Ritterbush and Bottjer, 2012). The original dataset was recorded by Whalen et al. (2020). SD = standard deviation, n = count.

	**Average**			**SD**			**Median**			
**Order**	**W (%)**	**1-Th (%)**	**U (%)**	**W (%)**	**1-Th (%)**	**U (%)**	**W (%)**	**1-Th (%)**	**U (%)**	**n**
**Agoniatitida**	0.430	0.722	0.291	0.156	0.146	0.145	0.433	0.748	0.267	113
**Goniatitida**	0.330	0.554	0.224	0.173	0.171	0.168	0.309	0.554	0.194	493
**Clymeniida**	0.322	0.811	0.489	0.143	0.122	0.151	0.297	0.831	0.501	66
**Prolecanitida**	0.611	0.806	0.195	0.196	0.094	0.147	0.596	0.814	0.176	58
**Ceratitida**	0.311	0.824	0.512	0.114	0.134	0.206	0.349	0.847	0.538	15

Table S10: Results of a non-parametric Dunn's test comparing the medians of each combination of Paleozoic ammonoid orders. Whorl expansion (W; used as a proxy for hydrostatic stability), 1-Th (compression ratio, used as a proxy for hydrodynamic stability), and umbilical exposure (U). Each coiling parameter (W, Th, U) represent percentages on a ternary diagram (see Ritterbush and Bottjer (2012). The original dataset was recorded by Whalen et al. (2020). Green numbers denote p-values lower than 0.05.

**Comparison**	**Dunn test (two-tailed p-values)**		
	**W (%)**	**1-Th (%)**	**U (%)**
**Agoniatitida vs Goniatitida**	8.19E-09	<1.00E-16	1.56E-05
**Agoniatitida vs Clymeniida**	6.23E-05	0.001	1.10E-08
**Agoniatitida vs Prolecanitida**	1.27E-05	0.003	1.84E-04
**Agoniatitida vs Ceratitida**	0.012	0.061	0.002
**Goniatitida vs Clymeniida**	0.884	<1.00E-16	<1.00E-16
**Goniatitida vs Prolecanitida**	<1.00E-16	<1.00E-16	0.269
**Goniatitida vs Ceratitida**	0.743	6.77E-08	8.98E-07
**Clymeniida vs Prolecanitida**	1.78E-13	0.927	2.22E-16
**Clymeniida vs Ceratitida**	0.815	0.936	0.866
**Prolecanitida vs Ceratitida**	1.53E-06	0.892	6.51E-07

Table S11: Average, standard deviation (SD), median, and count (n) of ammonoids for each age from the Carnian to Callovian (original dataset from Smith et al., 2014). Whorl expansion (W; used as a proxy for hydrostatic stability), 1-Th (compression ratio, used as a proxy for hydrodynamic stability), and umbilical exposure (U). Each coiling parameter (W, Th, U) represent percentages on a ternary diagram (see Ritterbush and Bottjer, 2012). P-values are reported for a two-tailed, Wilcoxon Rank Sum Test to distinguish medians between each age. Green numbers denote values lower than 0.05.

<table>
<tr><th rowspan="2">Period</th><th rowspan="2">Age</th><th colspan="3">Average</th><th colspan="3">SD</th><th colspan="3">Median</th><th rowspan="2">n</th><th colspan="3">Wilcoxon Rank Sum p-values</th></tr>
<tr><th>w (%)</th><th>1-Th (%)</th><th>U (%)</th><th>w (%)</th><th>1-Th (%)</th><th>U (%)</th><th>w (%)</th><th>1-Th (%)</th><th>U (%)</th><th>w (%)</th><th>1-Th (%)</th><th>U(%)</th></tr>
<tr><td rowspan="16">Jurassic</td><td rowspan="2">Callovian</td><td rowspan="2">0.333</td><td rowspan="2">0.719</td><td rowspan="2">0.386</td><td rowspan="2">0.138</td><td rowspan="2">0.153</td><td rowspan="2">0.145</td><td rowspan="2">0.334</td><td rowspan="2">0.745</td><td rowspan="2">0.376</td><td rowspan="2">36</td><td colspan="3"></td></tr>
<tr><td rowspan="2">0.8999</td><td rowspan="2">0.5061</td><td rowspan="2">0.5293</td></tr>
<tr><td rowspan="2">Bathonian</td><td rowspan="2">0.357</td><td rowspan="2">0.682</td><td rowspan="2">0.325</td><td rowspan="2">0.234</td><td rowspan="2">0.123</td><td rowspan="2">0.137</td><td rowspan="2">0.368</td><td rowspan="2">0.706</td><td rowspan="2">0.340</td><td rowspan="2">6</td></tr>
<tr><td rowspan="2">0.8946</td><td rowspan="2">0.1741</td><td rowspan="2">0.2617</td></tr>
<tr><td rowspan="2">Bajocian</td><td rowspan="2">0.343</td><td rowspan="2">0.750</td><td rowspan="2">0.407</td><td rowspan="2">0.188</td><td rowspan="2">0.151</td><td rowspan="2">0.175</td><td rowspan="2">0.332</td><td rowspan="2">0.791</td><td rowspan="2">0.415</td><td rowspan="2">98</td></tr>
<tr><td rowspan="2">0.0341</td><td rowspan="2">0.0066</td><td rowspan="2">0.9186</td></tr>
<tr><td rowspan="2">Aalenian</td><td rowspan="2">0.405</td><td rowspan="2">0.820</td><td rowspan="2">0.416</td><td rowspan="2">0.196</td><td rowspan="2">0.111</td><td rowspan="2">0.192</td><td rowspan="2">0.408</td><td rowspan="2">0.835</td><td rowspan="2">0.410</td><td rowspan="2">45</td></tr>
<tr><td rowspan="2">0.1114</td><td rowspan="2">0.0888</td><td rowspan="2">0.8653</td></tr>
<tr><td rowspan="2">Toarcian</td><td rowspan="2">0.371</td><td rowspan="2">0.787</td><td rowspan="2">0.416</td><td rowspan="2">0.175</td><td rowspan="2">0.114</td><td rowspan="2">0.167</td><td rowspan="2">0.343</td><td rowspan="2">0.818</td><td rowspan="2">0.425</td><td rowspan="2">83</td></tr>
<tr><td rowspan="2">0.8215</td><td rowspan="2">0.0229</td><td rowspan="2">0.1571</td></tr>
<tr><td rowspan="2">Pliensbachian</td><td rowspan="2">0.369</td><td rowspan="2">0.819</td><td rowspan="2">0.449</td><td rowspan="2">0.187</td><td rowspan="2">0.113</td><td rowspan="2">0.188</td><td rowspan="2">0.353</td><td rowspan="2">0.846</td><td rowspan="2">0.474</td><td rowspan="2">92</td></tr>
<tr><td rowspan="2">0.0565</td><td rowspan="2">0.5507</td><td rowspan="2">0.1800</td></tr>
<tr><td rowspan="2">Sinemurian</td><td rowspan="2">0.321</td><td rowspan="2">0.813</td><td rowspan="2">0.492</td><td rowspan="2">0.187</td><td rowspan="2">0.108</td><td rowspan="2">0.223</td><td rowspan="2">0.286</td><td rowspan="2">0.822</td><td rowspan="2">0.506</td><td rowspan="2">88</td></tr>
<tr><td rowspan="2">0.3654</td><td rowspan="2">0.0327</td><td rowspan="2">0.0655</td></tr>
<tr><td rowspan="2">Hettangian</td><td rowspan="2">0.288</td><td rowspan="2">0.861</td><td rowspan="2">0.573</td><td rowspan="2">0.140</td><td rowspan="2">0.094</td><td rowspan="2">0.193</td><td rowspan="2">0.262</td><td rowspan="2">0.878</td><td rowspan="2">0.598</td><td rowspan="2">44</td></tr>
<tr><td rowspan="2">0.0738</td><td rowspan="2">0.0004</td><td rowspan="2">0.0008</td></tr>
<tr><td rowspan="6">Triassic</td><td rowspan="2">Rhaetian</td><td rowspan="2">0.383</td><td rowspan="2">0.655</td><td rowspan="2">0.272</td><td rowspan="2">0.112</td><td rowspan="2">0.161</td><td rowspan="2">0.162</td><td rowspan="2">0.380</td><td rowspan="2">0.689</td><td rowspan="2">0.253</td><td rowspan="2">8</td></tr>
<tr><td rowspan="2">0.2200</td><td rowspan="2">0.0741</td><td rowspan="2">0.5906</td></tr>
<tr><td rowspan="2">Norian</td><td rowspan="2">0.452</td><td rowspan="2">0.760</td><td rowspan="2">0.308</td><td rowspan="2">0.162</td><td rowspan="2">0.100</td><td rowspan="2">0.179</td><td rowspan="2">0.460</td><td rowspan="2">0.790</td><td rowspan="2">0.302</td><td rowspan="2">81</td></tr>
<tr><td rowspan="2">0.0067</td><td rowspan="2">0.0901</td><td rowspan="2">0.6416</td></tr>
<tr><td rowspan="2">Carnian</td><td rowspan="2">0.384</td><td rowspan="2">0.705</td><td rowspan="2">0.321</td><td rowspan="2">0.163</td><td rowspan="2">0.164</td><td rowspan="2">0.178</td><td rowspan="2">0.350</td><td rowspan="2">0.759</td><td rowspan="2">0.312</td><td rowspan="2">76</td></tr>
<tr><td colspan="3"></td></tr>
<tr><td colspan="2">Jurassic</td><td>0.349</td><td>0.794</td><td>0.445</td><td>0.182</td><td>0.129</td><td>0.194</td><td>0.340</td><td>0.816</td><td>0.445</td><td>492</td><td rowspan="2">3.0E-05</td><td rowspan="2">1.1E-07</td><td rowspan="2">4.6E-13</td></tr>
<tr><td colspan="2">Triassic</td><td>0.417</td><td>0.730</td><td>0.312</td><td>0.164</td><td>0.140</td><td>0.178</td><td>0.407</td><td>0.776</td><td>0.302</td><td>165</td></tr>
</table>

Legend for Dataset S1: DOI: 10.5281/zenodo.6316035. This dataset consists of a .zip folder containing each component of the physical models used in the hydrodynamic restoration experiments (*Nautilus pompilius*, oxycone, serpenticone, and sphaerocone). Physical models 3D-printed in PETG allow the mass distribution to be calibrated with adjustable counterweights. The folder also contains sample footage of hydrodynamic restoration for the *Nautilus* model and the serpenticone model.

References

Peterman DJ, Barton CC, Yacobucci MM. 2019. The hydrostatics of Paleozoic ectocochleate cephalopods (Nautiloidea and Endoceratoidea) with implications for modes of life and early colonization of the pelagic zone. Palaeontologia Electronica 22.2.24A:1–29.

Peterman DJ, Ritterbush KA. 2022. Resurrecting extinct cephalopods with biomimetic robots to explore hydrodynamic stability, maneuverability, and physical constraints on life habits. Sci Rep 11287:1–16.

Ritterbush KA, Bottjer DJ. 2012. Westermann Morphospace displays ammonoid shell shape and hypothetical paleoecology. Paleobiology 38:424–46.

Smith PL, Longridge LM, Grey M, Zhang J, Liang B. 2014. From near extinction to recovery: Late Triassic to Middle Jurassic ammonoid shell geometry. Lethaia 47.

Whalen CD, Hull PM, Briggs DEG. 2020. Paleozoic ammonoid ecomorphometrics test ecospace availability as a driver of morphological diversification. Sci Adv 6.
